# Lower SARS-CoV-2–specific humoral immunity in people living with HIV-1 recovered from nonhospitalized COVID-19

**DOI:** 10.1172/jci.insight.158402

**Published:** 2022-11-08

**Authors:** Daniel J. Schuster, Shelly Karuna, Caroline Brackett, Martina Wesley, Shuying S. Li, Nathan Eisel, DeAnna Tenney, Sir’Tauria Hilliard, Nicole L. Yates, Jack R. Heptinstall, LaTonya D. Williams, Xiaoying Shen, Robert Rolfe, Robinson Cabello, Lu Zhang, Sheetal Sawant, Jiani Hu, April Kaur Randhawa, Ollivier Hyrien, John A. Hural, Lawrence Corey, Ian Frank, Georgia D. Tomaras, Kelly E. Seaton, HVTN 405/HPTN 1901 Study Team

**Affiliations:** 1 Center for Human Systems Immunology,; 2 Department of Surgery, and; 3 Department of Immunology, Duke University School of Medicine, Durham, North Carolina, USA.; 4 Vaccine and Infectious Disease Division, Fred Hutchinson Cancer Research Center, Seattle, Washington, USA.; 5 Division of Infectious Diseases, Department of Medicine, Duke University School of Medicine, Durham, North Carolina, USA.; 6 Asociación Civil Via Libre, Lima, Peru.; 7 Division of Infectious Disease, Perelman School of Medicine, University of Pennsylvania, Philadelphia, Pennsylvania, USA.; 8 Department of Molecular Genetics and Microbiology, Duke University School of Medicine, Durham, North Carolina, USA.; 9 The HVTN 405/HPTN 1901 Study Team are detailed in Supplemental Acknowledgments.

**Keywords:** AIDS/HIV, COVID-19, Immunoglobulins

## Abstract

People living with HIV-1 (PLWH) exhibit more rapid antibody decline following routine immunization and elevated baseline chronic inflammation than people without HIV-1 (PWOH), indicating potential for diminished humoral immunity during SARS-CoV-2 infection. Conflicting reports have emerged on the ability of PLWH to maintain humoral protection against SARS-CoV-2 coinfection during convalescence. It is unknown whether peak COVID-19 severity, along with HIV-1 infection status, associates with the quality and quantity of humoral immunity following recovery. Using a cross-sectional observational cohort from the United States and Peru, adults were enrolled 1–10 weeks after SARS-CoV-2 infection diagnosis or symptom resolution. Serum antibodies were analyzed for SARS-CoV-2–specific response rates, binding magnitudes, ACE2 receptor blocking, and antibody-dependent cellular phagocytosis. Overall, (a) PLWH exhibited a trend toward decreased magnitude of SARS-CoV-2–specific antibodies, despite modestly increased overall response rates when compared with PWOH; (b) PLWH recovered from symptomatic outpatient COVID-19 had comparatively diminished immune responses; and (c) PLWH lacked a corresponding increase in SARS-CoV-2 antibodies with increased COVID-19 severity when asymptomatic versus symptomatic outpatient disease was compared.

## Graphical abstract

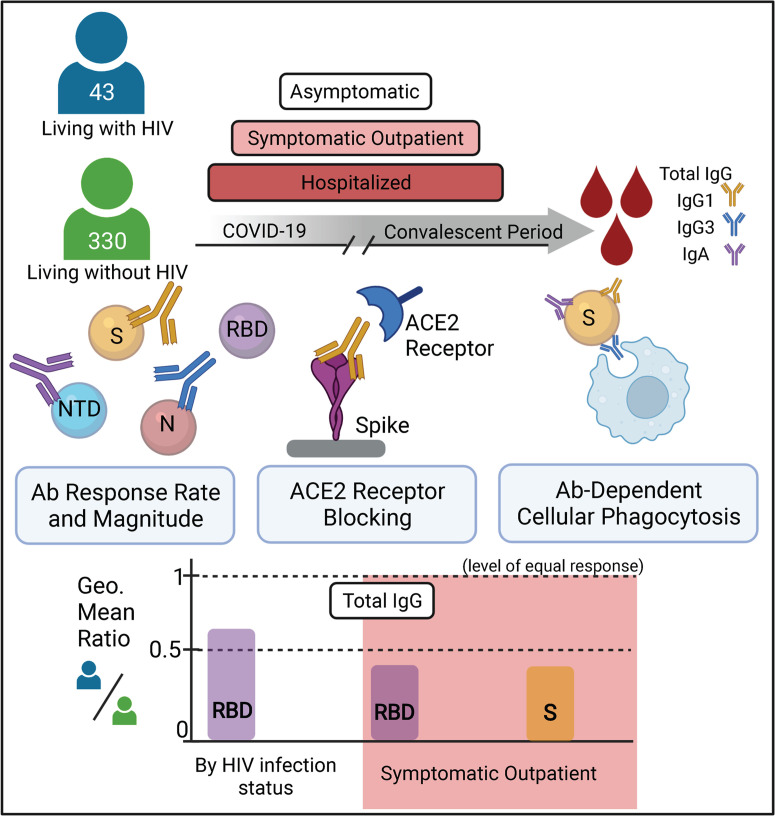

## Introduction

As the coronavirus disease 2019 (COVID-19) pandemic continues to impact people globally, tremendous efforts have focused on understanding humoral immune responses and protection from severe acute respiratory syndrome coronavirus 2 (SARS-CoV-2) infection. Studies have identified comorbidities such as hypertension, diabetes, and poorly controlled HIV-1, along with demographic characteristics including male sex assigned at birth and increased age, as risk factors for the development of severe COVID-19 (1, 2). With over 38 million people living with HIV-1 (PLWH) globally as of 2021, of whom an estimated 75% are on antiretroviral therapy (ART), key questions remain regarding humoral immune responses to SARS-CoV-2 in the convalescent period for this group (3).

Understanding the magnitude and functionality of SARS-CoV-2 humoral immune responses throughout the convalescent period is critical for vaccine design and implementation, particularly for individuals at high risk for severe COVID-19 (4). Antigenic targets include the spike trimer (typically stabilized with 2 or 6 prolines for experimental work), the ACE2-engaging receptor-binding domain (RBD), the N-terminal domain (NTD), and the viral RNA-binding nucleocapsid (N). Antibody isotype and subclass levels, ACE2 receptor blocking, and pseudotyped virus neutralization have been shown to track with acute COVID-19 severity (5–7). Furthermore, SARS-CoV-2 antigen–specific IgG and IgA antibodies have been detected up to 12 months after infection in people without HIV-1 (PWOH), indicating that robust and durable antibody titers can be generated to these viral antigens (8, 9). A recent study on PWOH has identified the correlation of vaccine-induced spike-specific IgG titers and neutralization with COVID-19 protection (10). However, discordant reports exist regarding the ability of PLWH coinfected with SARS-CoV-2 to maintain an effective humoral immune response into the convalescent period. Comparable SARS-CoV-2–specific total IgG titers 5–7 months after infection were reported for PLWH on ART and PWOH patients in the United Kingdom (11). PLWH in South Africa with well-controlled HIV-1 also demonstrated antibody kinetics, durability, and neutralization potency similar to those in PWOH (12). Similar antibody levels against the spike protein and nucleocapsid were reported in small PLWH cohorts in Japan (13) and the Netherlands (14), respectively. In contrast, other studies reported a marked decline of antibody responses within 2 months of SARS-CoV-2 infection among PLWH (15) with diminished seroconversion and shorter duration of antibody responses in comparison with PWOH (16).

There are well-documented challenges to generating and maintaining humoral responses to vaccinations and infection in the setting of HIV-1 infection that fuel the concern over durable SARS-CoV-2 protection after natural infection (17–22). Low CD4^+^ T cell counts (<300 cells/mL) in PLWH have previously been shown to correlate with impaired antibody titers following immunization with tetanus and diphtheria toxoid relative to PWOH (17). In a meta-analysis of duration of immunity following routine vaccinations, the rates of seroprotection at 2 and 5 years after vaccination were lower in PLWH compared with PWOH for hepatitis B, hepatitis A, measles, and *Streptococcus* *pneumoniae* (20). The ability of PLWH to maintain humoral protection following infection remains paramount to understanding the risk for reinfection, vaccine efficacy, and the need for additional vaccine boosters going forward.

We examined the SARS-CoV-2–specific humoral immune responses during the convalescent period using a large, multinational, adult cohort. Patients with recent SARS-CoV-2 infection were enrolled 1–8 weeks after symptom resolution if symptomatic or 2–10 weeks after diagnosis if asymptomatic and stratified by symptom severity to correlate with levels of total IgG, IgG subclasses, and IgA; ACE2 receptor blocking capacity; and antibody-dependent cellular phagocytosis. Together, these data shed light on the complex humoral milieu resulting from HIV-1 and SARS-CoV-2 coinfection, and highlight novel quantitative differences among PLWH recovered from symptomatic COVID-19 not requiring hospitalization.

## Results

### Participant characteristics.

We analyzed SARS-CoV-2–specific antibody responses by HIV-1 serostatus (43 PLWH, 330 PWOH). Median ages were 56 (IQR 35.5–69) and 53 (IQR 38–67) years, respectively. PLWH were more likely to currently smoke, or to have ever smoked, marijuana or currently smoke cigarettes (marijuana current: 18.6% vs. 3.9%, *P* < 0.001; marijuana ever: 46.5% vs. 23.9%, *P* = 0.003; cigarettes current: 23.3% vs. 4.5%, *P* < 0.001), and were more likely to identify as Black non-Hispanic (30.2% vs. 10.9%, *P* = 0.004) and to have been assigned male sex at birth (83.7% vs. 50.6%, *P* < 0.001) (Table 1). No significant differences between PLWH and PWOH were found for age, BMI category, chronic obstructive pulmonary disease/emphysema/asthma, peak COVID-19 severity, days from SARS-CoV-2 diagnosis (both overall and within each of the symptom severity categories), diabetes, hypertension, or status as prolonged viral shedders (Table 1 and Table 2).

### HIV-1 viral load, CD4 count, and ART.

Of the 43 PLWH participants, 42 reported currently taking ART, 24 of 27 (85.2%) with recently available viral load (VL) had levels lower than 50 copies/mL, and 24 of 26 (92.3%) with recently available CD4 counts had counts more than 300 cells/μL (Table 3).

### SARS-CoV-2 antibody response rates.

**Figure 1:**
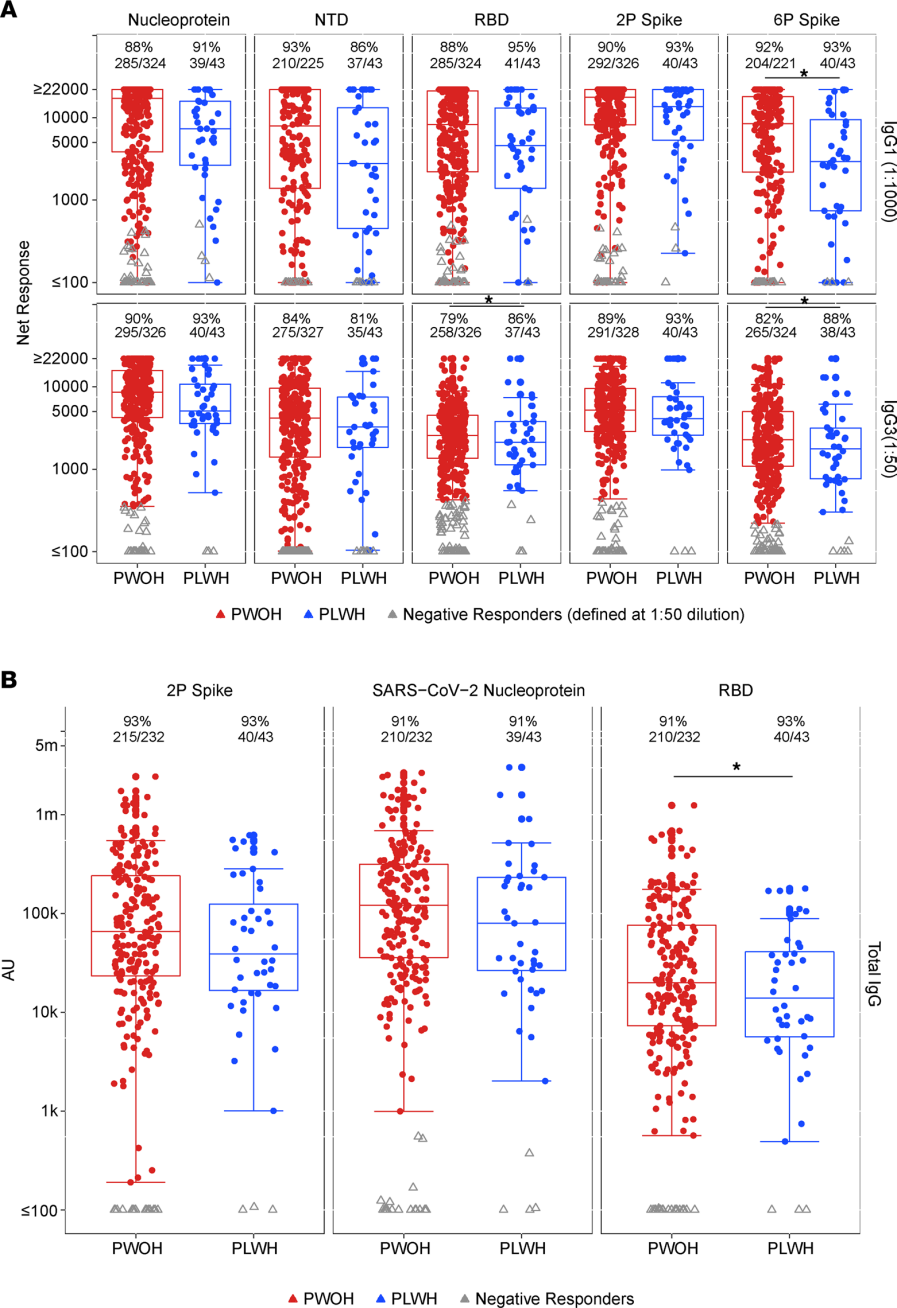
SARS-CoV-2–specific IgG1, IgG3, and total IgG response rates and magnitudes at enrollment by HIV serostatus. Response rates are shown above each box plot along with the number tested. RBD- and 6P spike–specific IgG3 response rates are significantly increased for PLWH (RBD: 86% vs. 79%, OR 2.81, *P* = 0.039; 6P spike: 88% vs. 82%, OR 3.23, *P* = 0.033). Colored dots, positive responders; red, PWOH; blue, PLWH; gray triangles, nonresponders. Box plots represent the distribution of magnitudes for the positive responders only. Prespecified IgG1 antigen-specific MFI positivity calls at 1:50 dilution were: RBD, 676; 2P spike, 1,967; 6P spike, 607; nucleoprotein, 1,666; NTD, 175. Instances of overlapping seropositive and seronegative responses at 1:1,000 dilution are below the positivity thresholds, and the positive responses at 1:50 are shown in Supplemental Figure 1. Response magnitude is shown as net response in mean fluorescence intensity (MFI) in **A** and as arbitrary units (AU) in **B**. 6P spike–specific IgG1 is significantly decreased for PLWH (geometric mean ratio [GMR] 0.63, *P* = 0.05, *q* = 0.138), and RBD-specific total IgG is significantly decreased for PLWH (GMR 0.63, *P* = 0.021, *q* = 0.093). Log-linear regression adjusting for peak COVID-19 symptom severity, diabetes, hypertension, chronic obstructive pulmonary disease (COPD)/emphysema/asthma, current and ever smoking, age, sex, BMI, race/ethnicity, region, and days since SARS-CoV-2 diagnosis was used. Asterisks and solid lines on top of response rates and box plots denote significant differences in response rate and response magnitude, respectively, at the *P* ≤ 0.05 and *q* ≤ 0.2 levels.

**Figure 2:**
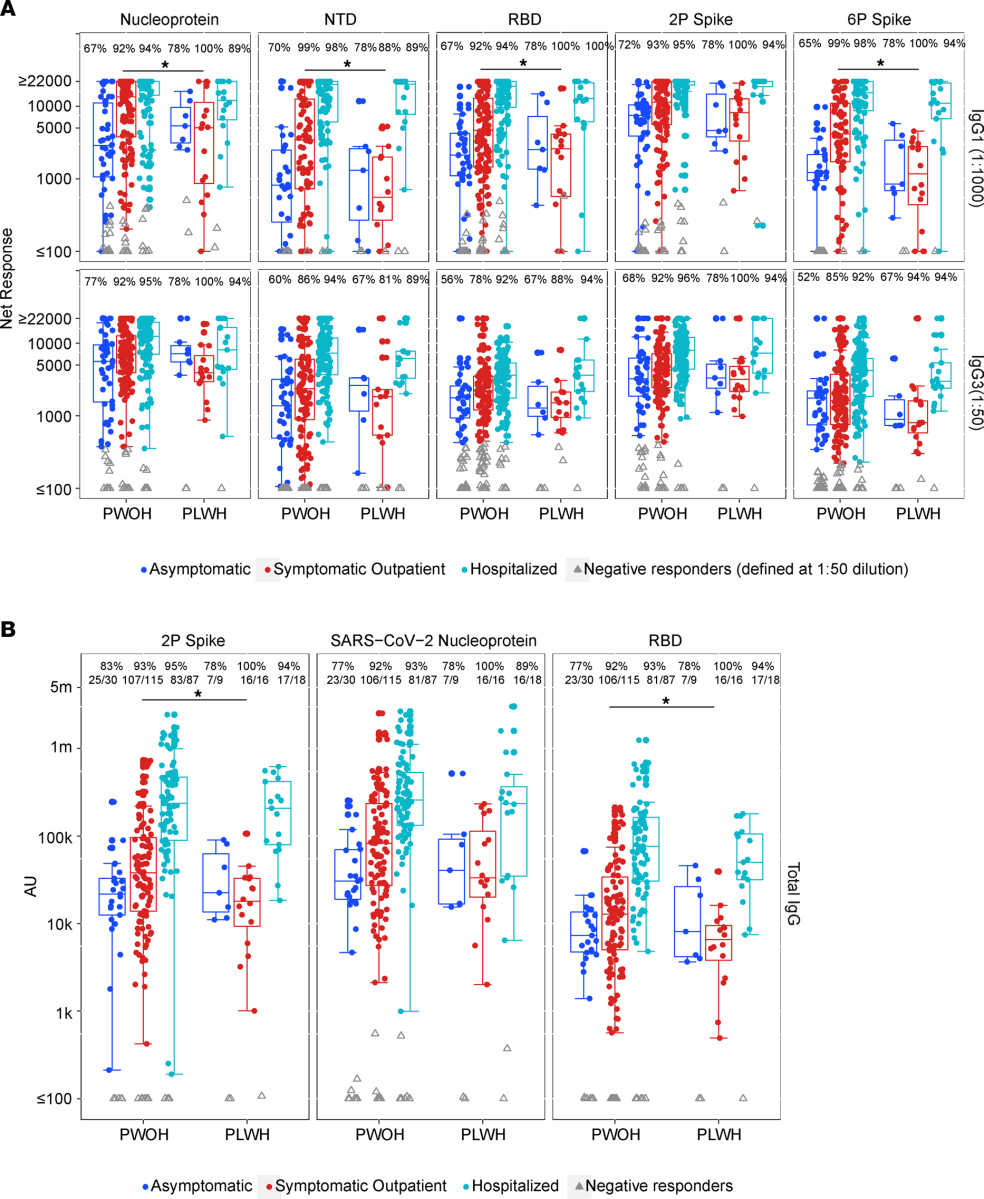
SARS-CoV-2–specific IgG1, IgG3, and total IgG response rates and magnitudes at enrollment by HIV serostatus and peak COVID-19 symptom severity. Response rates are shown at the top of each box plot. Colored dots/boxes designate peak symptom severity (blue, asymptomatic; red, symptomatic outpatient; teal, hospitalized). Gray triangles, nonresponders. Box plots represent the distribution for the positive responders only (number tested: PLWH IgG1 and IgG3 all antigens, *n* = 9 asymptomatic, *n* = 16 symptomatic outpatient, *n* = 18 hospitalized; PWOH IgG1 asymptomatic/symptomatic outpatient/hospitalized: N 64/130/130, NTD 40/82/103, RBD 63/131/130, 2P 64/133/129, 6P 40/79/102; PWOH IgG3 asymptomatic/symptomatic outpatient/hospitalized: N 64/131/131, NTD 65/131/131, RBD 64/131/131, 2P 65/132/131, 6P 63/130/131). Response magnitude is shown as net response in MFI in **A** and as AU in **B**. Prespecified IgG1 antigen-specific MFI positivity calls at 1:50 dilution were: RBD, 676; 2P spike, 1,967; 6P spike, 607; nucleoprotein, 1,666, NTD, 175. Instances of overlapping seropositive and seronegative responses at 1:1,000 dilution are below the positivity thresholds, and the positive responses at 1:50 are shown in Supplemental Figure 1. (**A**) IgG1 and IgG3. (**B**) Total IgG. PLWH recovered from symptomatic outpatient COVID-19 have significantly decreased response magnitudes for nucleoprotein-, NTD-, RBD-, and 6P spike–specific IgG1 (N: GMR 0.38, *P* = 0.004, *q* = 0.02; NTD: GMR 0.23, *P* < 0.001, *q* = 0.003; RBD: GMR 0.41, *P* = 0.005, *q* = 0.02; 6P spike: GMR 0.25, *P* < 0.001, *q* = 0.001) and 2P spike– and RBD-specific total IgG (2P: GMR 0.41, *P* =0.012, *q* = 0.054; RBD: GMR 0.43, *P* = 0.006, *q* = 0.053). Response rate differences are not present by HIV serostatus within symptom severity groups. Log-linear regression adjusting for peak COVID-19 symptom severity, diabetes, hypertension, COPD/emphysema/asthma, current and ever smoking, age, sex, BMI, race/ethnicity, region, and days since SARS-CoV-2 diagnosis was used. Asterisks and solid lines denote significant differences in response magnitude between PLWH and PWOH at *P* ≤ 0.05 and *q* ≤ 0.2 levels.

We examined whether response rates of SARS-CoV-2–specific antibodies (IgG1, IgG3, total IgG, and IgA) differed between PLWH and PWOH participants after adjusting for peak COVID-19 symptom severity, demographics, preexisting medical conditions, smoking history, region, and days since SARS-CoV-2 diagnosis. PLWH exhibited higher response rates and significantly higher odds ratios (ORs) of RBD- and 6P spike–specific IgG3 (79% vs. 86%, OR 2.81, *P* = 0.039, and 82% vs. 88%, OR 3.23, *P* = 0.033, respectively) (Figure 1 and Supplemental Table 1; supplemental material available online with this article; https://doi.org/10.1172/jci.insight.158402DS1). Further evaluation of response rate ORs stratified by peak COVID-19 symptom severity (asymptomatic, symptomatic outpatient, and hospitalized) failed to identify significant differences between the 2 groups (Figure 2, Supplemental Figure 3, and Supplemental Table 2). Within the PWOH group, an overall trend was present for increased response rate ORs with increased peak symptom severity (Supplemental Table 3). Symptomatic outpatient participants had significantly higher response rate ORs than asymptomatic participants across all antibody-antigen combinations, except for total IgG (Supplemental Table 3). Additionally, hospitalized participants had significantly increased response rate ORs compared with symptomatic outpatient participants for antigen-specific IgG3 and IgA (except for 6P spike). Within the PLWH group, symptomatic outpatient participants had significantly increased response rate ORs over asymptomatic participants for IgG1, IgG3 (except for NTD), total IgG (except for 2P spike), and IgA (except for RBD, nucleoprotein, and 2P spike) (Supplemental Table 4). Compiled response rates as a function of HIV-1 serostatus and peak COVID-19 symptom severity are depicted in Supplemental Figure 4.

### Magnitude of SARS-CoV-2 antibodies in PLWH.

We next assessed antibody response magnitudes to the SARS-CoV-2 antigen panel in PLWH as compared with PWOH. SARS-CoV-2 IgG3 and IgA are presented at a 1:50 dilution, which matches the dilution for the positivity cutoff. The magnitude of IgG1 is much higher, so the data are reported and compared at 1:1,000 dilution, within the linear range of the assay, to enable cross-group statistical comparisons. Response magnitudes among positive responders were overall lower (geometric mean ratio [GMR] < 1) in PLWH for all antibody-antigen pairs with only 6P spike–specific IgG1 (GMR 0.63, *P* = 0.05) and RBD-specific total IgG reaching statistical significance (GMR 0.63, *P* = 0.031) (Figure 1, Supplemental Figure 2, and Supplemental Table 1). Median magnitude of RBD-specific total IgG responses in WHO/National Institute for Biological Standards and Control (NIBSC) units was 378.18 versus 542.07 binding antibody units (BAU)/mL in PLWH and PWOH, respectively. IgG1-specific magnitude values of positive responders overlapping with negative responders were further explored as shown in Supplemental Figure 1 and confirmed to be overlapping only below the antigen-specific positivity cutoffs.

Further examination of the impact of peak COVID-19 symptom severity on response magnitudes identified a unique signature among symptomatic outpatient PLWH. RBD-, nucleoprotein-, NTD-, and 6P spike–specific IgG1 response magnitudes were significantly lower among PLWH than PWOH (GMR 0.41, *P* = 0.005; GMR 0.38, *P* = 0.004; GMR 0.23, *P* < 0.001; and GMR 0.25, *P* < 0.001, respectively) in addition to RBD- and 2P spike–specific total IgG (GMR 0.43, *P* = 0.006; and GMR 0.41, *P* = 0.012, respectively) (Figure 2, Supplemental Figure 3, and Supplemental Table 2). Median magnitude of IgG responses in WHO/NIBSC units was 178.83 versus 348.14 BAU/mL (RBD) and 161.68 versus 342.53 BAU/mL (2P spike), respectively.

Among PWOH, increased peak COVID-19 symptom severity resulted in an increased response magnitude overall (Figure 2 and Supplemental Table 3). In contrast, the PLWH response magnitude was similar between symptomatic outpatient and asymptomatic peak infection severity with the exception of nucleoprotein-specific IgA (GMR 2.05, *P* = 0.017; Figure 2, Supplemental Figure 3, and Supplemental Table 4). While symptomatic outpatient PLWH exhibited diminished antibody responses, response magnitude significantly increased in hospitalized versus symptomatic outpatient PLWH for all but 5 antibody-antigen pairs (Supplemental Table 4). Compiled response magnitudes as a function of HIV-1 serostatus and peak COVID-19 symptom severity are depicted in Supplemental Figure 5.

### ACE2 receptor blocking.

**Figure 3:**
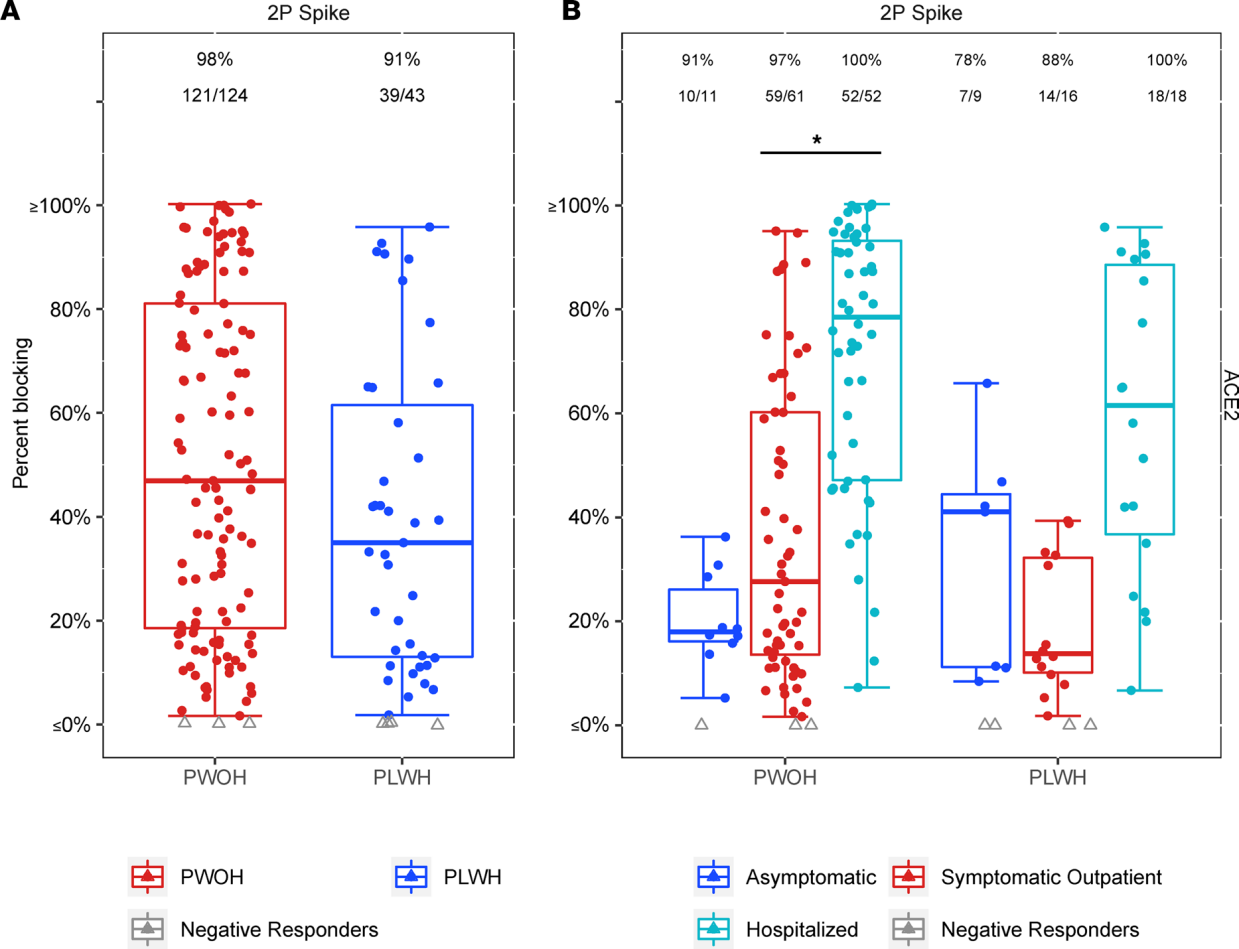
SARS-CoV-2 2P spike–specific percentage ACE2 receptor blocking by serum at enrollment as a function of HIV serostatus and peak COVID-19 symptom severity. Colored dots, positive responders; gray triangles, nonresponders. Box plots represent the distribution for positive responders only. (**A**) Response rates and the number tested are above each box plot (red, PWOH; blue, PLWH). (**B**) Response rates are above each box plot. Peak COVID-19 symptom severity is listed as: blue, asymptomatic; red, symptomatic outpatient; teal, hospitalized. No significant differences were detected between PLWH and PWOH. However, percentage blocking increased for hospitalized PWOH compared with symptomatic outpatient PWOH (OR 3.37, *P* = 0.005). Logistic regression adjusting for peak COVID-19 symptom severity, diabetes, hypertension, COPD/emphysema/asthma, current and ever smoking, age, sex, BMI, race/ethnicity, region, and days since SARS-CoV-2 diagnosis was used. Asterisks and solid lines denote significant differences at *P* ≤ 0.05 level. For within-group significant differences between peak COVID-19 symptom severities, see Supplemental Table 5.

The ability of SARS-CoV-2 spike–specific antibodies to block ACE2 receptor binding, considered to be the predominant mechanism of SARS-CoV-2 neutralization (23), was evaluated in samples from PLWH (*n* = 43) and PWOH (*n* = 124) participants. ACE2 receptor blocking was previously reported to correlate with live virus neutralization and is used as a surrogate to facilitate testing in a biosafety level 2 (BSL-2) as opposed to a BSL-3 laboratory (24). Response rates trended lower for PLWH when compared by HIV-1 status and when compared by peak COVID-19 symptom severity, but did not reach statistical significance (Figure 3 and Supplemental Table 5). Percent ACE2 blocking was not different by HIV-1 serostatus or by peak symptom severity. Among PLWH, no significant differences in response rates were observed with increasing COVID-19 severity, though a positive trend was present. Similar to the binding antibody responses, the PWOH group exhibited increased ACE2 blocking with increasing disease severity (hospitalized vs. symptomatic: OR 3.37, *P* = 0.005; Figure 3 and Supplemental Table 5).

### Association of VL and CD4 counts with antibody responses.

The association of VL and CD4 counts with antibody responses was next assessed. SARS-CoV-2–specific antibody responses demonstrated no statistically significant correlation with CD4 counts, and there were no significant differences in response rates when stratified by VL detection status, though subgroups were small (Supplemental Tables 6 and 7, respectively).

### Antibody-dependent cellular phagocytosis.

**Figure 4:**
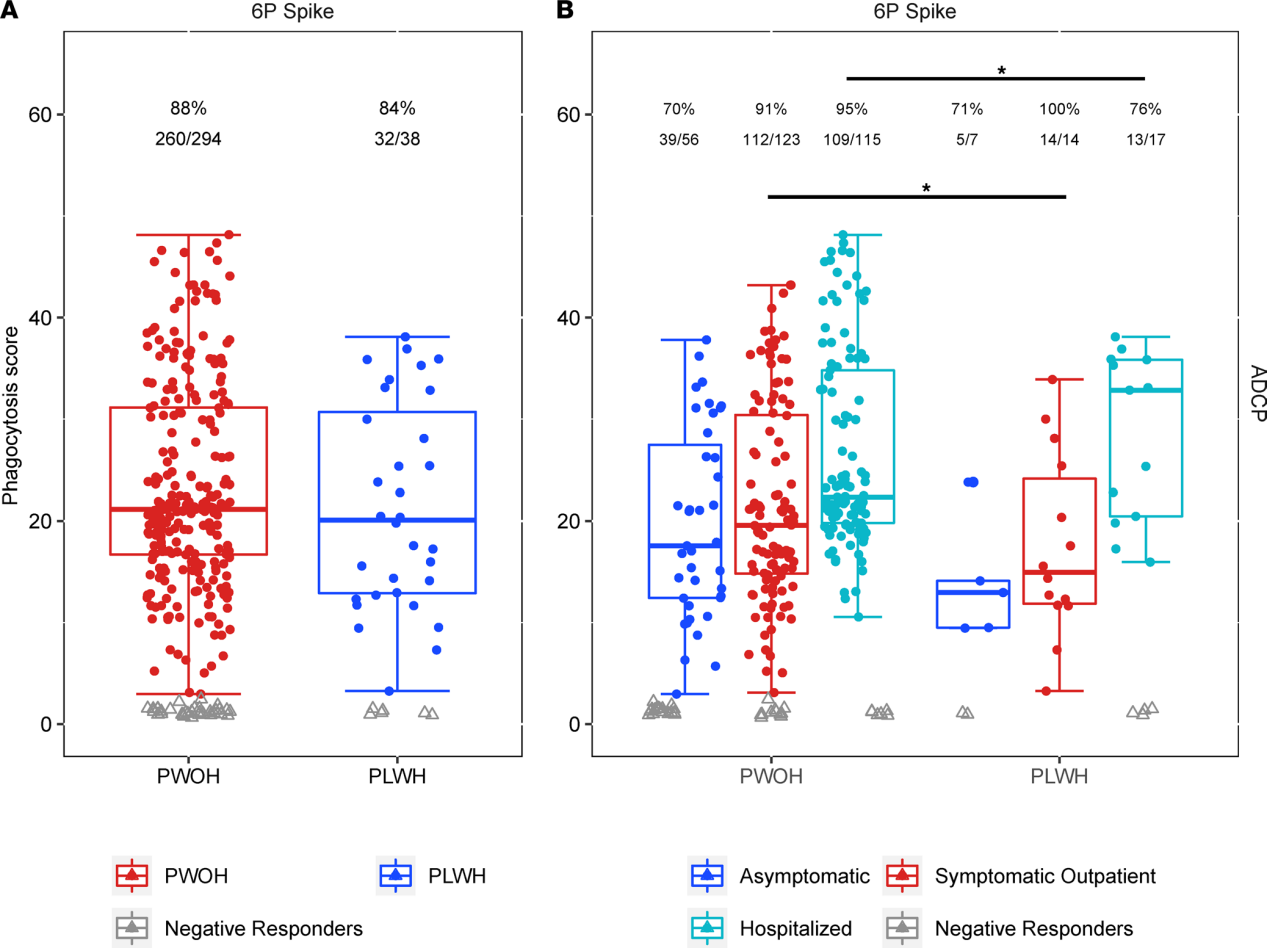
SARS-CoV-2 spike–specific ADCP by HIV serostatus and peak COVID-19 symptom severity. 6P spike is the antigenic target. Response rate is presented at the top of each box plot along with the number tested. (**A**) ADCP response rate and phagocytosis score as a function of HIV serostatus (red, PWOH; blue, PLWH; gray triangles, nonresponders). (**B**) ADCP response rate and phagocytosis score as a function of both HIV serostatus and peak COVID-19 symptom severity (blue, asymptomatic; red, symptomatic outpatient; teal, hospitalized). PLWH recovered from symptomatic outpatient COVID-19 have significantly decreased phagocytosis compared with PWOH (GMR 0.77, *P* = 0.045), while PLWH recovered from hospitalized COVID-19 have a significantly decreased response rate compared with PWOH (76% vs. 95%, OR 0.23, *P* = 0.039). Both PWOH and PLWH demonstrated significant response rate increases within their respective serostatus groups with increased severity from asymptomatic to symptomatic participants (PWOH: OR 4.44, *P* = 0.002; PLWH: OR 19.3, *P* = 0.049). For additional within-group significant differences between peak COVID-19 symptom severities, see Supplemental Table 8. Log-linear regression adjusting for peak COVID-19 symptom severity, diabetes, hypertension, COPD/emphysema/asthma, current and ever smoking, age, sex, BMI, race/ethnicity, region, and days since SARS-CoV-2 diagnosis was used. Asterisks and solid lines on top of response rate and box plots denote significant differences in response rate and response magnitude, respectively, between PLWH and PWOH at *P* ≤ 0.05 level.

PLWH are known to have alterations in total antibody Fc glycosylation, a key determinant of Fc effector functions such as antibody-dependent cellular phagocytosis (ADCP), even after achieving viral control on ART (25). ADCP is linked to decreased HIV-1 acquisition risk in a vaccine efficacy trial, suggesting its potential importance for protection from other viral etiologies (26). Indeed, significant differences in ADCP have been shown to exist between groups based on both COVID-19 symptom severity and comorbidities (27, 28). In order to assess the impact of HIV-1 on SARS-CoV-2–specific ADCP, samples (38 PLWH, 294 PWOH) were evaluated for cellular phagocytosis capacity. No significant differences were found in response rate or response magnitude (phagocytosis score) by HIV-1 serostatus alone (Figure 4 and Supplemental Table 8). When further stratified by peak COVID-19 symptom severity, hospitalized PLWH had a significantly lower response rate (OR 0.23, *P* = 0.039) while symptomatic outpatient PLWH had a significantly lower response magnitude (GMR 0.77, *P* = 0.045) than PWOH participants (Supplemental Table 8). Both PWOH and PLWH demonstrated significant response rate increases within their respective serostatus groups with increased severity from asymptomatic to symptomatic participants (PWOH: OR 4.44, *P* = 0.002; PLWH: OR 19.3, *P* = 0.049). However, only hospitalized versus symptomatic PWOH demonstrated a significantly increased response magnitude (GMR 1.21, *P* = 0.003).

### Results of sensitivity analysis.

Because of the limited number of PLWH in the study, the potential influence of comorbidities on the generation of humoral immunity, and the risk of overadjusting the model (29), we conducted a sensitivity analysis adjusting for a truncated list of covariates (COVID-19 severity, days since SARS-CoV-2 diagnosis, age, sex assigned at birth, and region). Results of the sensitivity analysis confirmed the following major findings in the primary model: (a) a trend toward decreased magnitude of SARS-CoV-2–specific antibodies in PLWH, despite modestly increased overall response rates when compared with PWOH; (b) diminished immune responses in symptomatic outpatient PLWH when compared with PWOH; and (c) the absence of a rise in SARS-CoV-2–specific humoral immune responses from asymptomatic to symptomatic outpatient SARS-CoV-2 infection in PLWH. Additional minor differences in the statistical significance of individual immune responses between the primary analysis and the sensitivity analysis are presented in Supplemental Table 9.

## Discussion

Characterizing SARS-CoV-2–specific humoral immune responses in people living with HIV-1 is a critical component of assessing potential protection from reinfection and informing an understanding of immune responses to preventative vaccines. Spike- and RBD-specific IgG titers, along with neutralization, were recently identified as correlates of decreased infection risk and increased vaccine efficacy (10). Previous studies in PLWH, not involving SARS-CoV-2 infection, have noted immune responses distinct from those in PWOH, suggesting that humoral immunity after recovery from COVID-19 may also be impaired. PLWH have more rapid declines in antibody levels following routine vaccinations (20). Additionally, in PLWH prevaccine levels of soluble inflammatory markers have been associated with blunted immune responses to hepatitis A and B virus vaccines (30), and lymphoid tissue fibrosis, a pathological hallmark of chronic HIV replication, is associated with blunted responses to yellow fever vaccine (31). Together, these studies suggest an altered immune milieu among PLWH and motivated the current investigation.

In this study we performed an in-depth exploration of SARS-CoV-2–specific total IgG, IgG subclasses, IgA, and antibody effector functions including ACE2 blocking and ADCP in COVID-19–convalescent PLWH and PWOH. This study also uniquely analyzed humoral immune responses by HIV-1 serostatus and peak COVID-19 symptom severity while controlling for several other potential confounders, including diabetes, hypertension, smoking history, and BMI. To our knowledge, IgG subclass–specific SARS-CoV-2 responses were not previously reported in the setting of HIV-1 and SARS-CoV-2 coinfection. Using this study design and analytic approach illuminated several differences between PLWH and PWOH.

Analyzing humoral immune responses by HIV-1 serostatus alone revealed few statistically significant differences, though SARS-CoV-2–specific response magnitudes in PLWH trended lower overall. These results suggest that PLWH are capable of mounting a robust immune response to SARS-CoV-2 infection. Whereas response magnitudes of total IgG, IgG1, IgG3, and IgA increased among PWOH with increased COVID-19 symptom severity, in agreement with Luo et al. (7), those magnitudes among PLWH were not significantly increased in symptomatic outpatient compared with asymptomatic cases. Yates et al. noted the importance of considering IgG subclasses as well, as RBD- and S1-specific IgG3-biased responses significantly increased with symptom severity (6). Additionally, IgG1 and total IgG response magnitudes toward SARS-CoV-2 antigens were decreased for symptomatic outpatient PLWH compared with symptomatic outpatient PWOH, and more similar to those of asymptomatic PLWH. The similarity between PLWH recovered from asymptomatic and symptomatic outpatient SARS-CoV-2 infection may reflect a higher threshold requirement for antigen stimulation among PLWH. Aberrant CD4/CD8 ratios, an elevated baseline inflammatory state, or lymphoid fibrosis may contribute to this phenomenon. Interestingly, both PWOH and PLWH who had required hospitalization for their COVID-19 symptoms demonstrated similarly robust humoral responses.

Prior studies of humoral immune responses to SARS-CoV-2 in PLWH have yielded inconsistent observations. Alrubayyi et al. found similar rates (95.8% vs. 93.5%) and magnitudes of total IgG response to the S1 spike and N proteins between PLWH and PWOH at a median of 146 and 181 days, respectively, after symptom onset (11). Similarly, Snyman et al. found no differences by HIV-1 serostatus among a sub-Saharan African cohort in time to seroconversion (RBD-specific total IgM, IgG, and IgA), titers out to 3 months after enrollment, and live virus microneutralization (12). In contrast, Spinelli et al. found a significant decrease (by 53%) among PLWH in SARS-CoV-2 RBD-specific total IgG with samples collected a median of 2 months after diagnosis (15). Liu et al. found lower IgG seroconversion rates during acute infection (55.5% vs. 88.1%) and a significantly decreased IgG seropositivity 7–10 months later (12% vs. 33%) in PLWH compared with PWOH, though only 83.3% and 72.2% of the PLWH were on ART and virally suppressed, respectively (16). Samples from our study were collected earlier in the convalescent period (PLWH: median 56 days [IQR 35.5–69]; PWOH: 53 days [IQR 38–67]). As antibody titers wane over time, it is possible that samples analyzed in the Alrubayyi et al. study were too remote from the time of infection to detect significant differences. Recent work by Sandberg et al. analyzing a cohort of PWOH found S- and N-specific IgG levels to be increased with symptom severity during the acute phase of infection, but that difference disappeared in the late convalescent period (5–9 months later) (32). It is possible that the decrease among PLWH found in the Spinelli et al. study was driven by IgG1, the dominant IgG subclass, among symptomatic outpatient cases as a majority of participants in that study experienced only mild symptoms. Important differences may exist in the study populations, as Snyman et al. and Alrubayyi et al. included PLWH well controlled or virally suppressed on ART while Spinelli et al. and Liu et al. included participants with both virologically well-controlled and poorly controlled HIV-1 with lower rates of ART use (11, 12, 15, 16). Inclusion criteria for our study were not restricted to well-controlled HIV-1, and approximately one-third of the individuals lacked recently available VL and CD4 data, limiting our analysis. Given the paucity of data from HIV-1 and SARS-CoV-2 coinfection, future studies are needed to confirm the trend seen here among asymptomatic and symptomatic outpatient individuals.

The ability of SARS-CoV-2–specific antibodies to block spike binding to the ACE2 receptor in a pseudo-neutralization assay and to engage effector cells of the innate immune system in an ADCP assay offers insight into the functional attributes of the humoral immune response (33). We found comparable ACE2 receptor blocking rates and magnitudes, independent of HIV-1 infection status and peak COVID-19 severity. These results align with the neutralization assays of Alrubayyi et al. (11) and Snyman et al. (12) but differ from those of Spinelli et al. (15). While Spinelli et al. controlled for age, sex, and days since infection, similar to our primary and sensitivity analyses, differences in CD4/CD8 ratios, not captured in either study, may be a driver of divergent results (15). Avelino-Silva et al. demonstrated that a direct relationship exists between increased CD4/CD8 ratios and neutralizing antibody titers to a yellow fever vaccine given to PLWH (21). Another important difference between these studies is the method used to assess neutralization — Spinelli et al. employed a thin-film interferometry immunoassay specific to IgG, while the other studies used pseudotyped and live-virus assays (11, 12, 15). Prior work reported increased ADCP capacity for anti–SARS-CoV-2 antibodies among hospitalized compared with nonhospitalized patients and among those with preexisting comorbidities (27, 28). Our study found no significant difference in ADCP response rate or phagocytosis magnitude by HIV-1 serostatus alone. Together, these results suggest that the relatively diminished IgG response magnitudes maintain their specificity and ability to elicit phagocytosis. This may be accounted for by the similar median durations since infection for PLWH and PWOH, and the time available for plasma cells to produce potent antibodies.

There are several limitations to our study. By the nature of this convalescent cross-sectional study, results are subject to survivorship bias. As all measurements were only from enrollment, no antibody kinetics can be inferred. While the median duration from diagnosis to enrollment was nearly 2 months, the earliest time points may not fully represent the convalescent period. Direct viral detection testing was reported by the participants, and therefore viral samples were not available for sequencing. Given the enrollment dates, SARS-CoV-2 infection with D614G is assumed, and antigens used in assays were wild type (D614). The PLWH sample size was relatively small, and recent VL and CD4 data were only available from the medical records of a subset of participants. Durations of HIV infection or ART and recent CD8 counts were not collected at time of enrollment. Cellular analyses were not included in this study, thus limiting the scope of the conclusions to the array of antibody specificities, forms, and functions analyzed. This study was not powered to assess the impact of comorbidities on immune response differences. Given the impact of controlling for different variables in the 2 statistical models and an incomplete understanding of the effect of smoking and comorbidities on humoral immunity, we highlighted similarities between the primary model and the sensitivity analysis as they are likely to be the most robust and reproducible. While the trends in response magnitudes were consistent across antigens, conformational or epitope-specific differences in the assays may account for differences in reaching statistical significance.

In conclusion, we believe our results demonstrate that ART-treated PLWH coinfected with SARS-CoV-2 maintain a comparable humoral immune response into the convalescent period as compared with PWOH, with the novel exception of those recovered from outpatient symptomatic disease. Additional work remains to understand the etiology of that discrepancy and its implications for vaccine efficacy and protection from future SARS-CoV-2 challenges.

## Methods

### Study conduct and clinical trial information.

Details of study conduct and clinical trial information were previously reported in Karuna et al. (34). Briefly, participants recovered from SARS-CoV-2 infection were enrolled between May and October 2020 in the HIV Vaccine Trials Network (HVTN) 405/HIV Prevention Trials Network (HPTN) 1901 observational cohort study (ClinicalTrials.gov NCT04403880) led by the COVID-19 Prevention Trials Network (CoVPN). US (*n* = 195) and Peruvian (*n* = 178) participants, including 43 PLWH, were stratified by peak symptom severity (asymptomatic, symptomatic outpatient, and hospitalized) and by age (18–55 years and 55+ years). Peak symptom severities were self-reported as asymptomatic if no symptoms were present at the time of diagnosis through recovery, symptomatic if any symptoms were reported, and hospitalized if hospitalized due to COVID-19. Detailed information on demographics, comorbidities, and habits were collected at time of enrollment along with self-reported date of positive direct viral detection testing (i.e., antigen or molecular test). HIV-1 status, CD4 counts, and HIV-1 viral loads were reported by the enrolling clinics from participants’ health records. This study included samples only from the enrollment visit. All assays were conducted in compliance with Good Clinical Laboratory Practice guidelines for consistency and reproducibility.

### Antibody measurements.

SARS-CoV-2–specific IgG1, IgG3, and IgA were measured by Binding Antibody Multiplex Assay as previously described (35–38) with modifications. Briefly, antigens were bound to NeutrAvidin-coupled fluorescent microspheres (MagPlex, Luminex Corp.) via a biotinylated rabbit anti-6x His-tag antibody to directionally orient the F(ab′)_2_ arms outward. Prepared microspheres were incubated with human sera (IgG1 at 1:50, 1:1,000, 1:10,000, and 1:25,000; IgG3 and IgA at 1:50 and 1:250) and controls diluted in assay diluent for 2 hours, shaking at 750 rpm and 22°C. Subsequently, a mouse anti-human IgG1 (BioLegend, clone 12G8G11) or IgG3 (Invitrogen, clone HP6047) followed by goat anti-mouse IgG-PE (Southern Biotech, catalog 1030-09) was used to detect bound IgG1 and IgG3, respectively. Goat anti-human IgA-PE (Jackson ImmunoResearch, catalog 109-006-011) was used to detect IgA. IgA samples were IgG-depleted before testing using a protein G MultiTrap plate (GE Healthcare Bio Sciences AB, Uppsala, Sweden). Assay plates were read using a Bio-Plex 200 System (Bio-Rad). Sixty-eight SARS-CoV-2–seronegative samples, collected prior to November 2019, were tested at a 1:50 dilution to establish isotype- and antigen-specific positivity cutoffs (95th percentile and ≥100 net mean fluorescence intensity [MFI]) (BioIVT). Antigen panel components are listed in Supplemental Table 10. All samples, controls, and standards were assayed in duplicate, and the mean value is reported. Negative controls and uncoupled microspheres were included in each assay to ensure specificity. Levey-Jennings charts were used to track antigen performance across assays. Response calls were made with serum at a 1:50 dilution to increase sensitivity, while response magnitudes were reported at 1:1,000 for IgG1 to increase the number of samples within the linear range.

### Antibody-dependent cellular phagocytosis.

The ADCP assay was modeled after prior work (26, 33) with modifications. Briefly, quantification of ADCP was performed by covalently binding 6P spike (HexaPro) (39) to NeutrAvidin fluorescent beads (Thermo Fisher Scientific) and forming immune complexes by incubation with 1:50 diluted serum. This dilution was chosen from a 6-place 5-fold titration series starting from 1:10. HexaPro was used based on its more highly stabilized trimer conformation than 2P spike (39). Monoclonal antibodies CV23 IgG1 and CV30 IgG1 (40) and CR3022 IgG1 served as positive controls, while CH65 IgG1 served as a negative control (41). Immune complexes were incubated with THP-1 cells (ATCC), and cellular fluorescence was measured using a BD LSR Fortessa (BD Biosciences). Seventy-two SARS-CoV-2–seronegative samples were tested at a 1:50 dilution and processed to establish the positivity cutoff (95th percentile and 3 times the median) (BioIVT). ADCP scores were calculated as (MFI × frequency of phagocytosis-positive cells)/(MFI × frequency of bead-positive cells in a PBS control well).

### MSD four-plex SARS-CoV-2 IgG binding assay.

SARS-CoV-2 spike–, S1 RBD–, and nucleocapsid–specific IgG in serum samples was quantitatively measured using the V-PLEX SARS-CoV-2 384 Panel 1 (IgG) kit as previously described (42), according to the manufacturer’s instructions (Meso Scale Diagnostics [MSD]). Briefly, precoated MULTI-SPOT 384-Well plates were blocked (Blocker A solution) for 1 hour at 20°C–26°C. Plates were washed with MSD Wash Buffer, and samples were added to the plate, tested in duplicate at 1:500, 1:10,000, 1:200,000, and 1:4,000,000 dilutions. Plates were washed after 4 hours, and binding was detected using a mouse anti-human IgG conjugated to MSD SULFO-TAG. Following addition of MSD GOLD Read Buffer B, plates were read on a MESO SECTOR S 600MM instrument. Sixty-six SARS-CoV-2–seronegative serum samples were tested at a 1:500 dilution and processed to establish the positivity cutoff (mean + 3 SD) (BioIVT). Magnitude of binding in arbitrary units per milliliter (AU/mL) was calculated at each sample dilution by backfitting to a 7-place calibration curve run in duplicate on each plate. The median AU/mL from all dilutions in the linear range of the curve were used to calculate the final AU/mL for each sample. Conversion to WHO/NIBSC international standard units of binding antibody units (BAU/mL) was calculated with MSD units (AU/mL) × a conversion factor for reference standard 1 (lot A00V004) (0.00236, 0.0272, and 0.00901 for nucleocapsid, RBD, and spike, respectively) available through MSD.

### MSD ACE2 blocking assay.

Antibodies that block binding of SARS-CoV-2 spike to ACE2 were quantitatively measured using the V-PLEX SARS-CoV-2 Panel 2 (ACE2) kit according to the manufacturer’s instructions (MSD). Briefly, SARS-CoV-2 spike–coated MULTI-SPOT 96-Well plates were blocked and washed as above. Samples were tested in duplicate at a dilution of 1:250. Samples were selected based on RBD-specific IgG1 response magnitudes in a semi-random way using the following approach: (a) samples with positive responses passing quality control were evenly divided into top, middle, and bottom thirds and “high blank” (blank MFI > 5,000), (b) random numbers were assigned, and (c) 25 samples were selected from each tertile along with all 24 from the “high blank” group. Sixty-eight additional samples (blinded to our laboratory) were added to include all PLWH samples. A 7-place calibration curve and blank well were run in duplicate on each plate as well as a positive control mutant ACE2 protein (4-fold, 4-place dilution starting at 6 μg/mL). Samples were incubated with human ACE2 protein conjugated to MSD SULFO-TAG, washed, and read as above. Seventy-two SARS-CoV-2–seronegative samples were tested at a 1:250 dilution and processed to establish the positivity cutoff (mean + 3 SD, after truncation of all negative values to zero). Percentage blocking for samples was calculated from the 7-place calibration curve using the following equation: (1 – (sample electrochemiluminescent [ECL] signal mean – calibrator 1 ECL signal mean)/(blank well ECL signal mean – calibrator 1 ECL signal mean)) × 100.

### Statistical methods.

Participant characteristics were compared between PLWH and PWOH using χ^2^ test for categorical variables and 2-tailed *t* test for continuous variables. Comparisons of days since SARS-CoV-2 diagnosis across peak symptom severity groups within PLWH and PWOH were made using 1-way ANOVA tests. Positive responders for SARS-CoV-2 antigens were determined as described above for each assay type. Response rates and magnitudes between PLWH and PWOH were compared using the Firth logistic regression in accordance with Heinze and Schemper (43), log-linear (for IgG1, IgG3, total IgG, IgA, and ADCP response magnitudes), and logistic (for percentage ACE2 blocking) regressions, adjusting for all potential confounders (COVID-19 severity, diabetes, hypertension, chronic obstructive pulmonary disease/emphysema/asthma, current and ever cigarette/marijuana smoking, age, sex, BMI, race/ethnicity, region, and days since SARS-CoV-2 diagnosis) in a primary analysis. We also performed a sensitivity analysis in which we ran the same regression models described for the primary analysis but adjusting for only COVID-19 severity, age, sex, region, and days since SARS-CoV-2 diagnosis. Comparisons were further carried out between PLWH and PWOH stratifying by peak COVID-19 severity and between peak COVID-19 severity levels within PLWH and PWOH using the regression models described above plus an interaction between HIV-1 serostatus and COVID-19 severity. *Q* values were calculated for multiple comparisons involving multiple antigens in each type of response measure using the Benjamini and Hochberg method (44). *P* values ≤ 0.05 and *q* values ≤ 0.2 were considered significant. Spearman correlations of CD4 count with SARS-CoV-2–specific antibody responses were calculated among PLWH with available CD4 count data. Response rates were compared between PLWH with detectable and undetectable viral load using χ^2^ test. All analyses were performed using R (R Core Team [2020], Vienna, Austria).

### Study approval.

IRB approval was granted by a central IRB (Advarra IRB) and, as applicable, by individual clinical research sites’ IRBs. All participants provided written informed consent prior to participation.

## Author contributions

KES, NLY, XS, and GDT conceived and designed the research plan. SK, LC, IF, RC, JAH, AKR, and HVTN 405/HPTN 1901 Study Team designed the clinical study and/or enrolled participants. CB, MW, SH, JRH, DJS, DT, NE, and LDW designed assays and/or performed experiments. JRH, LDW, NLY, GDT, and KES supervised research. DJS, SK, IF, SSL, GDT, and KES wrote and edited the manuscript. LZ, SS, SSL, JH, and OH analyzed data, and RR and IF contributed to data interpretation. All authors reviewed the manuscript.

## Supplementary Material

Supplemental data

## Figures and Tables

**Table 2:**
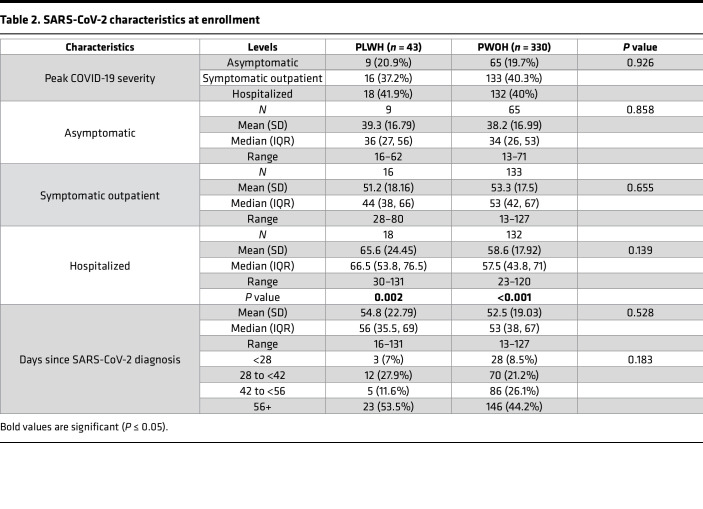
SARS-CoV-2 characteristics at enrollment

**Table 1:**
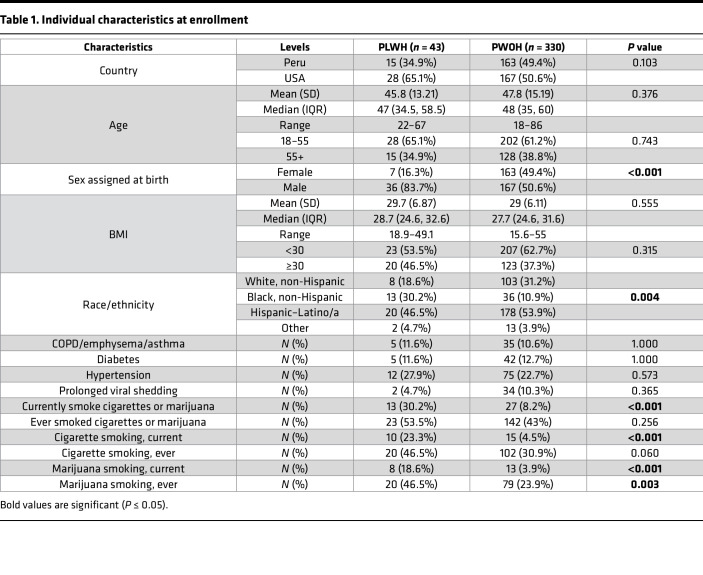
Individual characteristics at enrollment

**Table 3:**
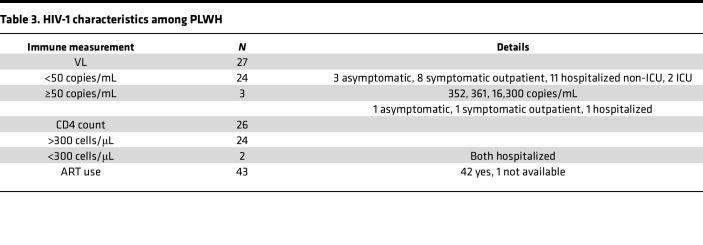
HIV-1 characteristics among PLWH

## Version 1 09/22/2022

In-Press Preview

## Version 2 11/08/2022

Electronic publication

## References

[1] Ambrosioni J (2021). Overview of SARS-CoV-2 infection in adults living with HIV. Lancet HIV.

[2] Fung M, Babik JM (2021). COVID-19 in immunocompromised hosts: what we know so far. Clin Infect Dis.

[3] https://www.unaids.org/en/resources/fact-sheet.

[4] Galipeau Y (2020). Humoral responses and serological assays in SARS-CoV-2 infections. Front Immunol.

[5] Atyeo C (2020). Distinct early serological signatures track with SARS-CoV-2 survival. Immunity.

[6] Yates JL (2021). Serological analysis reveals an imbalanced IgG subclass composition associated with COVID-19 disease severity. Cell Rep Med.

[7] Luo H (2021). The characterization of disease severity associated IgG subclasses response in COVID-19 patients. Front Immunol.

[8] Crawford KHD (2021). Dynamics of neutralizing antibody titers in the months after severe acute respiratory syndrome coronavirus 2 infection. J Infect Dis.

[9] Li C (2021). Twelve-month specific IgG response to SARS-CoV-2 receptor-binding domain among COVID-19 convalescent plasma donors in Wuhan. Nat Commun.

[10] Gilbert PB (2022). Immune correlates analysis of the mRNA-1273 COVID-19 vaccine efficacy trial. Science.

[11] Alrubayyi A (2021). Characterization of humoral and SARS-CoV-2 specific T cell responses in people living with HIV. Nat Commun.

[12] Snyman J (2022). Similar antibody responses against SARS-CoV-2 in HIV uninfected and infected individuals on antiretroviral therapy during the first South African infection wave. Clin Infect Dis.

[13] Yamamoto S (2021). Antibody response to SARS-CoV-2 in people living with HIV. J Microbiol Immunol Infect.

[14] Verburgh ML (2022). Similar risk of severe acute respiratory syndrome coronavirus infection and similar nucleocapsid antibody levels in people with well-controlled HIV and a comparable cohort of people without HIV. J Infect Dis.

[15] Spinelli MA (2021). SARS-CoV-2 seroprevalence, and IgG concentration and pseudovirus neutralising antibody titres after infection, compared by HIV status: a matched case-control observational study. Lancet HIV.

[16] Liu Y (2021). People living with HIV easily lose their immune response to SARS-CoV-2: result from a cohort of COVID-19 cases in Wuhan, China. BMC Infect Dis.

[17] Kroon FP (1995). Antibody response to diphtheria, tetanus, and poliomyelitis vaccines in relation to the number of CD4^+^ T lymphocytes in adults infected with human immunodeficiency virus. Clin Infect Dis.

[18] Kroon FP (1999). Immunoglobulin G (IgG) subclass distribution and IgG1 avidity of antibodies in human immunodeficiency virus-infected individuals after revaccination with tetanus toxoid. Clin Diagn Lab Immunol.

[19] Van den Berg R (2009). Non-responsiveness to hepatitis B vaccination in HIV seropositive patients; possible causes and solutions. AIDS Rev.

[20] Kerneis S (2014). Long-term immune responses to vaccination in HIV-infected patients: a systematic review and meta-analysis. Clin Infect Dis.

[21] Avelino-Silva VI (2016). CD4/CD8 ratio and KT ratio predict yellow fever vaccine immunogenicity in HIV-infected patients. PLoS Negl Trop Dis.

[22] Van Woudenbergh E (2020). HIV is associated with modified humoral immune responses in the setting of HIV/TB coinfection. mSphere.

[23] Johnson M (2020). Evaluation of a novel multiplexed assay for determining IgG levels and functional activity to SARS-CoV-2. J Clin Virol.

[24] Tan CW (2020). A SARS-CoV-2 surrogate virus neutralization test based on antibody-mediated blockage of ACE2-spike protein-protein interaction. Nat Biotechnol.

[25] Irvine EB, Alter G (2020). Understanding the role of antibody glycosylation through the lens of severe viral and bacterial diseases. Glycobiology.

[26] Neidich SD (2019). Antibody Fc effector functions and IgG3 associate with decreased HIV-1 risk. J Clin Invest.

[27] Yu KK (2021). Comorbid illnesses are associated with altered adaptive immune responses to SARS-CoV-2. JCI Insight.

[28] Adeniji OS (2021). COVID-19 severity is associated with differential antibody Fc-mediated innate immune functions. mBio.

[29] Breslow N (1982). Design and analysis of case-control studies. Annu Rev Public Health.

[30] Shive CL (2018). Pre-vaccine plasma levels of soluble inflammatory indices negatively predict responses to HAV, HBV, and tetanus vaccines in HCV and HIV infection. Vaccine.

[31] Kityo C (2018). Lymphoid tissue fibrosis is associated with impaired vaccine responses. J Clin Invest.

[32] Sandberg JT (2021). SARS-CoV-2-specific humoral and cellular immunity persists through 9 months irrespective of COVID-19 severity at hospitalisation. Clin Transl Immunology.

[33] Tay MZ (2019). Antibody-dependent cellular phagocytosis in antiviral immune responses. Front Immunol.

[34] Karuna S (2021). Neutralizing antibody responses over time in demographically and clinically diverse individuals recovered from SARS-CoV-2 infection in the United States and Peru: a cohort study. PLoS Med.

[35] Tomaras GD (2008). Initial B-cell responses to transmitted human immunodeficiency virus type 1: virion-binding immunoglobulin M (IgM) and IgG antibodies followed by plasma anti-gp41 antibodies with ineffective control of initial viremia. J Virol.

[36] Fouda GG (2011). HIV-specific functional antibody responses in breast milk mirror those in plasma and are primarily mediated by IgG antibodies. J Virol.

[37] Yates NL (2011). Multiple HIV-1-specific IgG3 responses decline during acute HIV-1: implications for detection of incident HIV infection. AIDS.

[38] Liu P (2011). Dynamic antibody specificities and virion concentrations in circulating immune complexes in acute to chronic HIV-1 infection. J Virol.

[39] Schaub JM (2021). Expression and characterization of SARS-CoV-2 spike proteins. Nat Protoc.

[40] Seydoux E (2020). Analysis of a SARS-CoV-2-infected individual reveals development of potent neutralizing antibodies with limited somatic mutation. Immunity.

[41] Whittle JR (2011). Broadly neutralizing human antibody that recognizes the receptor-binding pocket of influenza virus hemagglutinin. Proc Natl Acad Sci U S A.

[42] Pino M (2021). A yeast expressed RBD-based SARS-CoV-2 vaccine formulated with 3M-052-alum adjuvant promotes protective efficacy in non-human primates. Sci Immunol.

[43] Heinze G, Schemper M (2002). A solution to the problem of separation in logistic regression. Stat Med.

[44] Benjamini Y, Hochberg Y (1995). Controlling the false discovery rate: a practical and powerful approach to multiple testing. J R Stat Soc B.

