# Supplementary material for: Lower SARS-CoV-2–specific humoral immunity in people living with HIV-1 recovered from nonhospitalized COVID-19
Source: JCI Insight. 2022 Nov 8;7(21):e158402. doi: 10.1172/jci.insight.158402 (PMC9675463; doi:10.1172/jci.insight.158402)
Supplement: Supplemental data [file jciinsight-7-158402-s128.pdf]

Supplemental Figure S1: SARS-CoV-2-specific IgG1 Response Magnitudes at Enrollment by Responder Status Relative to the Positivity Cutoff IgG1 magnitudes are shown for positive (red) and negative (blue) responders in Figure 1A. Pre-specified positivity cutoffs are displayed for each antigen at the top of each panel and with a red dashed line. Clear separation is present at the 1:50 dilution used to make the positivity calls. At the 1:1000 dilution, the visual overlap with non-responders is below the antigen specific cutoff.

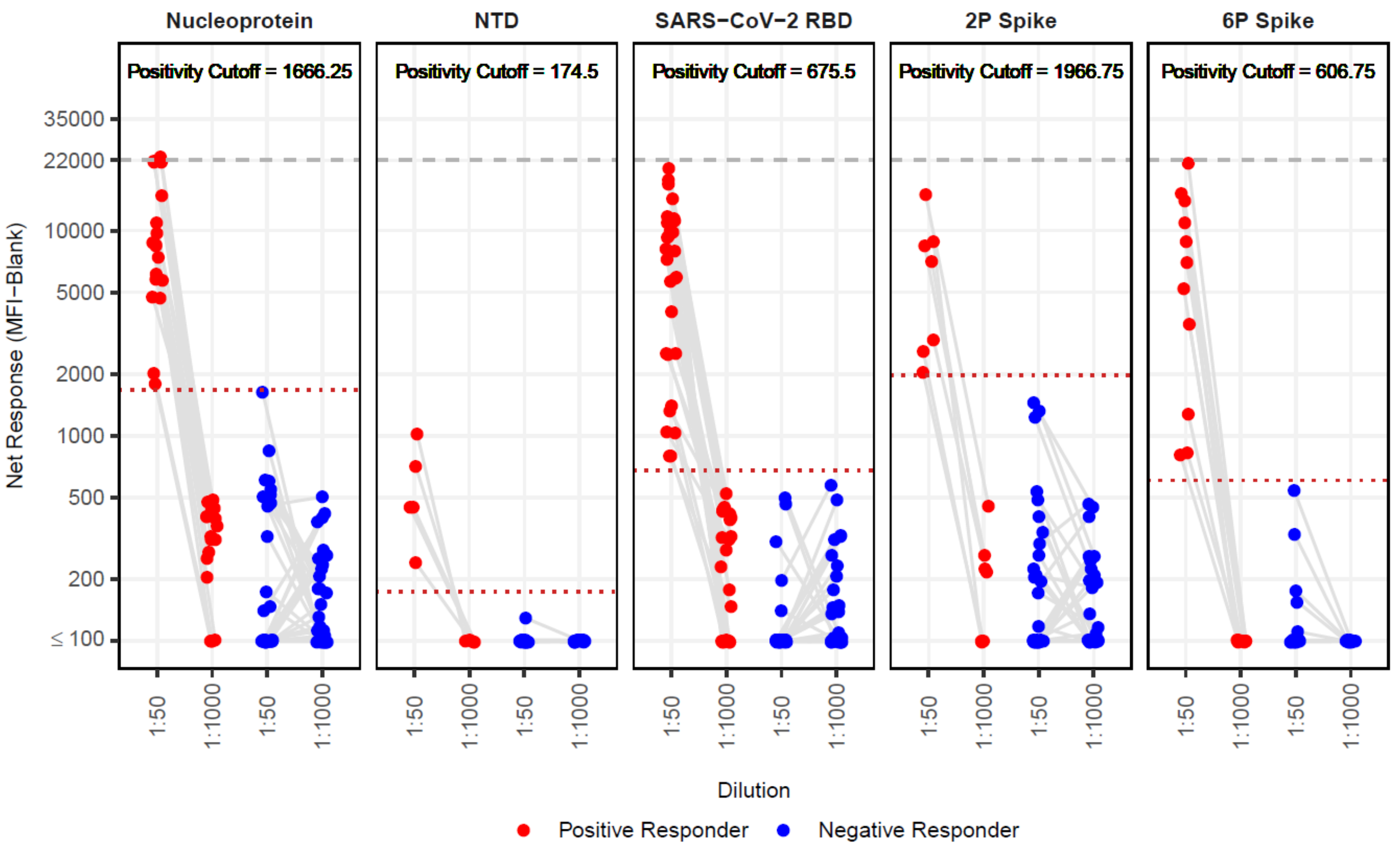

Supplemental Figure S2: SARS-CoV-2-specific IgA Response Rates and Magnitudes at Enrollment by HIV Serostatus

SARS-CoV-2-specific antigens are listed at the top of each column. Response rates are shown at the top of each box plot along with the number tested. Positive responders are depicted by colored dots (PWOH in red, PLWH in blue), and non-responders are depicted by gray triangles. Boxplots represent the distribution of magnitudes for the positive responders only. Response magnitude is shown as Net Response in mean fluorescent intensity (MFI). Log-linear regression adjusting for peak COVID-19 symptom severity, diabetes, hypertensions, COPD/emphysema/asthma, current and ever smoking, age, sex, BMI race/ethnicity, region, and days since SARS-CoV-2 diagnosis was used. Significant differences in the response rates and magnitudes were not detected at the p≤0.05 and q≤0.2 levels.

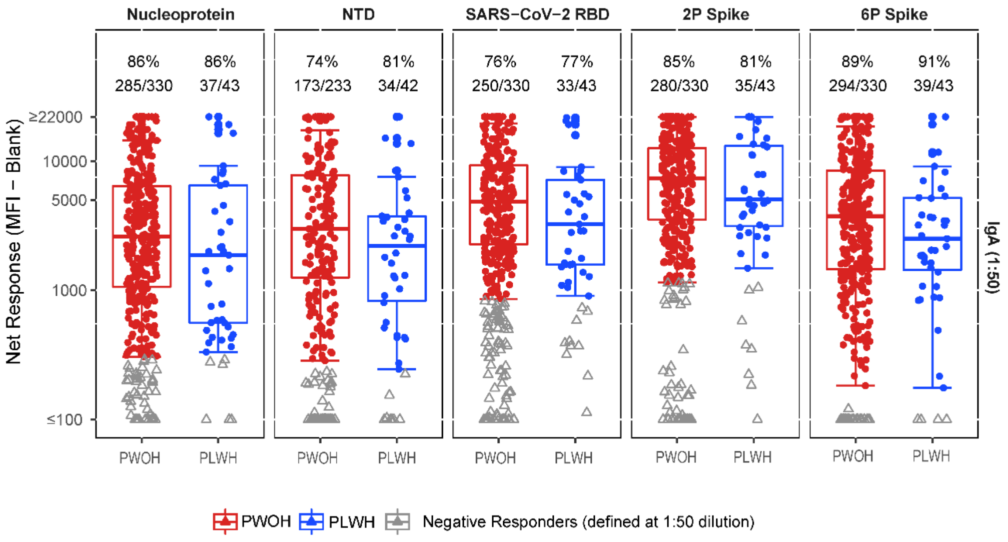

Supplemental Figure S3: SARS-CoV-2-specific IgA Response Rates and Magnitudes at Enrollment by HIV Serostatus and Peak COVID-19 Symptom Severity

SARS-CoV-2-specific antigens are listed at the top of each column. Response rates are shown at the top of each box-and-whisker plot along with the number tested. PWOH are shown on the left of each subpanel, and PLWH are shown on the right. Colored dots/boxes designate peak symptom severity (asymptomatic = blue, symptomatic outpatient = red, hospitalized = teal). Gray triangles = non-responders. Boxplots represent the distribution for the positive responders only (number tested - PLWH IgA Asymptomatic/Symptomatic Outpatient/Hospitalized: N 9/16/18, NTD 9/15/18, RBD 9/16/18, 2P 9/16/18, 6P 9/16/18; PWOH IgA Asymptomatic/Symptomatic Outpatient/ Hospitalized: N 65/133/132 , NTD 55/75/103, RBD 65/133/132, 2P 65/133/132, 6P 65/133/132). Response magnitude is shown as Net Response in mean fluorescent intensity (MFI). Log-linear regression adjusting for peak COVID-19 symptom severity, diabetes, hypertensions, COPD/emphysema/asthma, current and ever smoking, age, sex, BMI race/ethnicity, region, and days since SARS-CoV-2 diagnosis was used. Significant differences in the response rates and

magnitudes were not detected at the p≤0.05 and q≤0.2 levels. For within group significances between peak COVID-19 symptom severities, see Tables S5 and S6.

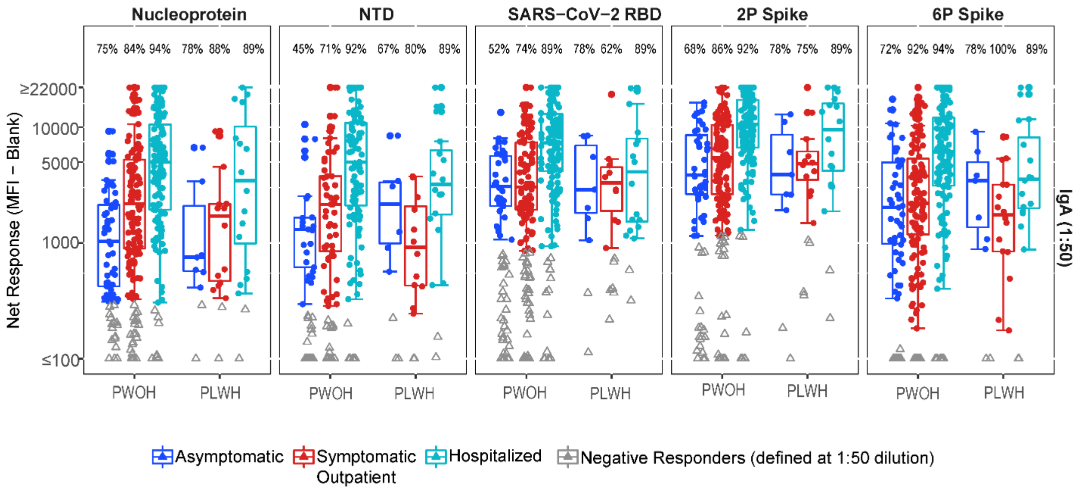

Supplemental Figure S4: Polar Plots of Response Rates to SARS-CoV-2 Antigens at Enrollment by HIV Serostatus and Peak COVID-19 Severity

Each plot represents a specific HIV serostatus and peak COVID-19 symptom severity. Isotypes/subclasses, ACE2 blocking, and ADCP are shown around the perimeter. Response rate (%) towards antigens are represented by colored rays. All participant data is included.

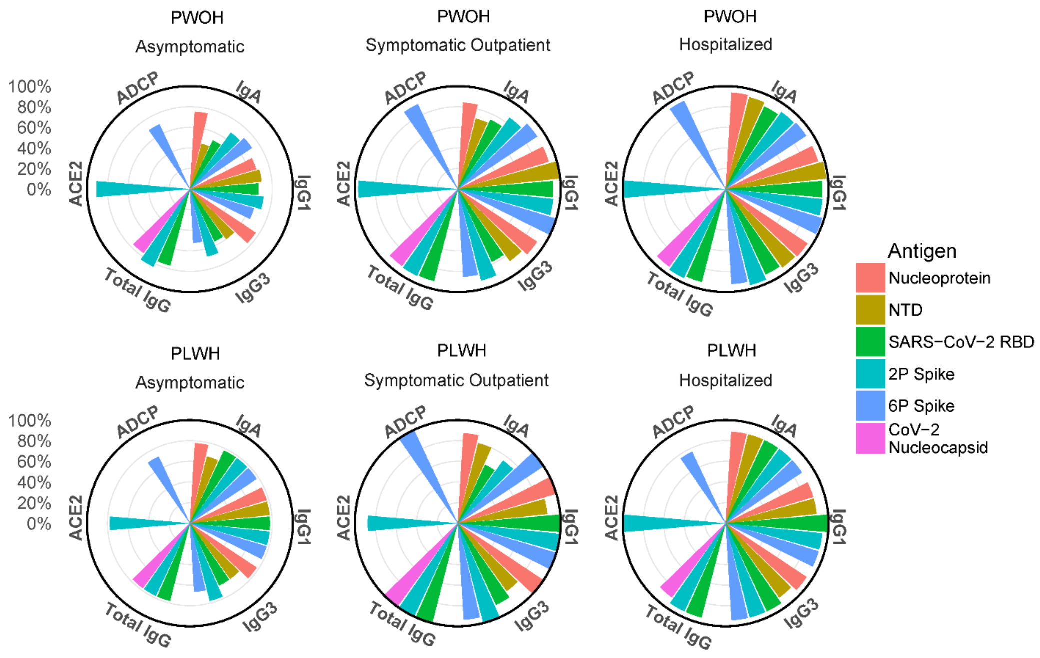

Supplemental Figure S5: Polar Plots of Mean Percentile of Response Magnitudes to SARS-CoV-2 Antigens at Enrollment by HIV Serostatus and COVID-19 Severity

Each plot represents a specific HIV serostatus and peak COVID-19 symptom severity. Isotypes/subclasses, ACE2 blocking, and ADCP are shown around the perimeter. Group mean percentile response magnitudes towards antigens are represented by colored rays. Response magnitudes were first ranked and then scaled from 0 to 1 for each antigen-antibody combination. Red circles at the 0.4 ring emphasize differences between asymptomatic and symptomatic outpatient group responses. All participant data is included.

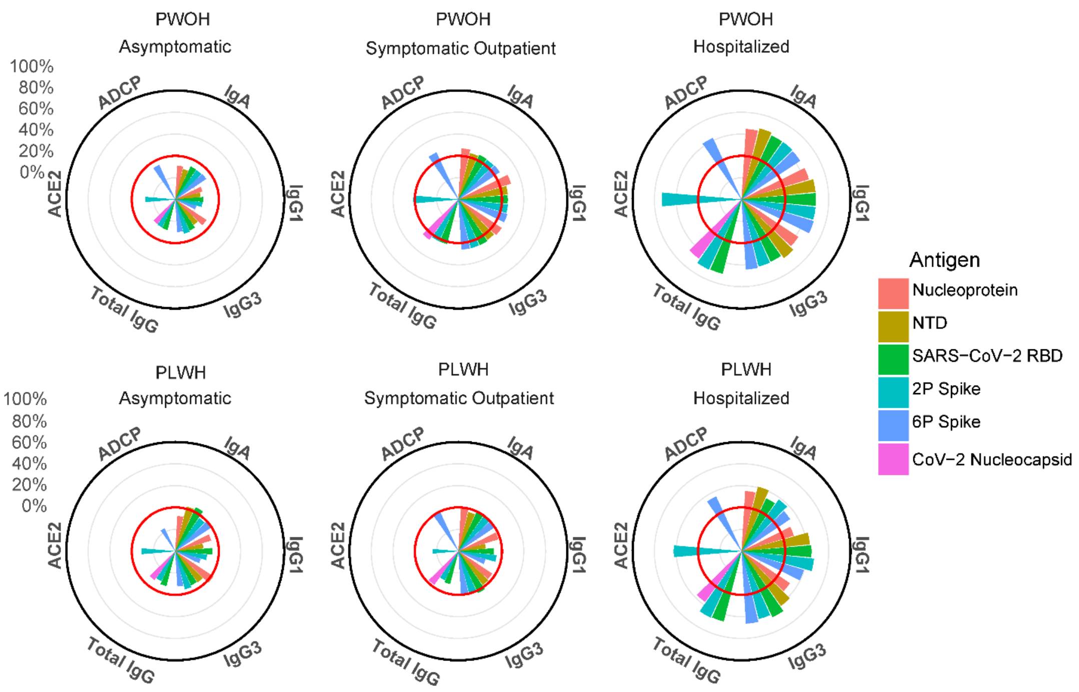

Supplemental Table 1: Association of HIV status with binding antibody responses adjusting for COVID-19 severity, demographics, pre-existing medical conditions, smoking history, region, and days since SARS-CoV-2 diagnosis

Isotype	Antigen	Response Rate				Response Magnitudes among Positive Responders			
		OR[*]	95% CI	P-value	Q-value	GMR[+]	95% CI	P-value	Q-value
IgG1	SARS-CoV-2 RBD	3.65	[0.97, 20.51]	0.057	0.245	0.75	[0.5, 1.14]	0.184	0.307
	SARS-CoV-2 Nucl	2.41	[0.75, 9.42]	0.147	0.245	0.65	[0.42, 1.01]	0.055	0.138
	SARS-CoV-2 2P Spike	2.18	[0.64, 9.66]	0.226	0.282	0.99	[0.72, 1.37]	0.969	0.969
	SARS-CoV-2-NTD	0.69	[0.16, 3.1]	0.616	0.616	0.73	[0.43, 1.25]	0.252	0.315
	SARS-CoV-2 6P Spike	3.6	[0.67, 25.58]	0.141	0.245	**0.63**	**[0.4, 1]**	**0.05**	**0.138**
IgG3	SARS-CoV-2 RBD	**2.81**	**[1.05, 8.68]**	**0.039**	**0.097**	0.95	[0.71, 1.29]	0.758	0.809
	SARS-CoV-2 Nucl	2.25	[0.66, 9.99]	0.207	0.259	0.88	[0.64, 1.21]	0.429	0.809
	SARS-CoV-2 2P Spike	2.88	[0.84, 12.87]	0.097	0.162	0.97	[0.73, 1.28]	0.809	0.809
	SARS-CoV-2-NTD	1.46	[0.55, 4.27]	0.459	0.459	0.89	[0.58, 1.34]	0.573	0.809
	SARS-CoV-2 6P Spike	**3.23**	**[1.09, 11.39]**	**0.033**	**0.097**	0.8	[0.57, 1.12]	0.195	0.809
IgG	CoV-2 Nucleoprotein	2.21	[0.64, 9.41]	0.221	0.332	0.64	[0.4, 1.04]	0.071	0.106
	CoV-2 RBD	2.59	[0.7, 12.47]	0.163	0.332	**0.63**	**[0.42, 0.96]**	**0.031**	**0.093**
	CoV-2 2P Spike	1.83	[0.48, 8.89]	0.394	0.394	0.71	[0.45, 1.14]	0.155	0.155

IgA	SARS-CoV-2 RBD	1.12	[0.46, 2.89]	0.803	0.927	0.8	[0.6, 1.05]	0.112	0.281
	SARS-CoV-2 Nucl	0.83	[0.3, 2.52]	0.73	0.927	0.9	[0.63, 1.3]	0.586	0.645
	SARS-CoV-2 2P Spike	0.96	[0.38, 2.6]	0.927	0.927	0.94	[0.73, 1.21]	0.645	0.645
	SARS-CoV-2-NTD	1.79	[0.65, 5.46]	0.27	0.833	0.86	[0.59, 1.26]	0.445	0.645
	SARS-CoV-2 6P Spike	1.78	[0.57, 6.69]	0.333	0.833	0.76	[0.54, 1.05]	0.098	0.281

*: OR=Odds Ratio, +: GMR=Geometric Mean Ratio; Nucl is short for Nucleoprotein; Bold values are significant (p-value≤0.05 and q-value≤0.2)

Supplemental Table 2: Association of HIV status with binding antibody responses stratified by COVID-19 severity adjusting for demographics, pre-existing medical conditions, smoking history, region, and days since SARS-CoV-2 diagnosis

			Response Rate				Response Magnitudes			
**Isotype**	**Antigen**	**Comparison**	**OR**	**95% CI**	**P-value**	**Q-value**	**GMR**	**95% CI**	**P-value**	**Q-value**
IgG1	SARS-CoV-2 RBD	PLWH vs PWOH in Asymptomatic	1.59	[0.26, 9.69]	0.612	0.921	2.7	[1.02, 7.15]	0.046	0.138
		PLWH vs PWOH in Symptomatic	5.66	[0.28, 112.66]	0.256	0.73	**0.41**	**[0.22, 0.77]**	**0.005**	**0.02**
		PLWH vs PWOH in Hospitalized	5.18	[0.27, 100.16]	0.276	0.73	0.84	[0.47, 1.53]	0.577	0.665
	SARS-CoV-2 Nucleoprotein	PLWH vs PWOH in Asymptomatic	1.98	[0.32, 12.47]	0.465	0.921	2.17	[0.79, 5.98]	0.133	0.284
		PLWH vs PWOH in Symptomatic	8.3	[0.42, 165.7]	0.166	0.73	**0.38**	**[0.2, 0.74]**	**0.004**	**0.02**
		PLWH vs PWOH in Hospitalized	0.93	[0.16, 5.36]	0.936	0.936	0.7	[0.37, 1.32]	0.266	0.467
	SARS-CoV-2 2P Spike	PLWH vs PWOH in Asymptomatic	1.33	[0.22, 8.17]	0.755	0.936	1.86	[0.88, 3.93]	0.103	0.257

Isotype	Antigen	Comparison	Response Rate OR	95% CI	P-value	Q-value	Response Magnitudes GMR	95% CI	P-value	Q-value
		PLWH vs PWOH in Symptomatic	4.91	[0.25, 94.87]	0.292	0.73	0.8	[0.49, 1.3]	0.372	0.503
		PLWH vs PWOH in Hospitalized	1.43	[0.2, 10.53]	0.723	0.936	0.97	[0.61, 1.54]	0.888	0.951
	SARS-CoV-2-NTD	PLWH vs PWOH in Asymptomatic	2	[0.22, 18.48]	0.54	0.921	1.68	[0.5, 5.66]	0.402	0.503
		PLWH vs PWOH in Symptomatic	0.13	[0.01, 1.48]	0.101	0.73	**0.23**	**[0.1, 0.51]**	**<0.001**	**0.003**
		PLWH vs PWOH in Hospitalized	0.78	[0.09, 6.92]	0.82	0.936	1.4	[0.67, 2.91]	0.373	0.503
	SARS-CoV-2 6P Spike	PLWH vs PWOH in Asymptomatic	4.32	[0.5, 37.11]	0.183	0.73	1.81	[0.62, 5.3]	0.28	0.467
		PLWH vs PWOH in Symptomatic	1.19	[0.04, 31.63]	0.916	0.936	**0.25**	**[0.13, 0.5]**	**<0.001**	**0.001**
		PLWH vs PWOH in Hospitalized	1.89	[0.16, 22.18]	0.614	0.921	1.01	[0.54, 1.9]	0.975	0.975
IgG3	SARS-CoV-2 RBD	PLWH vs PWOH in Asymptomatic	2.44	[0.47, 12.55]	0.286	0.66	0.92	[0.44, 1.94]	0.83	0.89

			Response Rate				Response Magnitudes			
**Isotype**	**Antigen**	**Comparison**	**OR**	**95% CI**	**P-value**	**Q-value**	**GMR**	**95% CI**	**P-value**	**Q-value**
		PLWH vs PWOH in Symptomatic	2.81	[0.59, 13.42]	0.194	0.66	0.69	[0.43, 1.1]	0.122	0.452
		PLWH vs PWOH in Hospitalized	2.3	[0.34, 15.66]	0.396	0.66	1.25	[0.82, 1.92]	0.3	0.643
	SARS-CoV-2 Nucleoprotein	PLWH vs PWOH in Asymptomatic	1.05	[0.17, 6.54]	0.958	0.964	1.9	[0.89, 4.02]	0.095	0.452
		PLWH vs PWOH in Symptomatic	6.67	[0.32, 138.28]	0.22	0.66	0.68	[0.41, 1.11]	0.12	0.452
		PLWH vs PWOH in Hospitalized	1.7	[0.21, 13.75]	0.618	0.843	0.85	[0.53, 1.35]	0.484	0.726
	SARS-CoV-2 2P Spike	PLWH vs PWOH in Asymptomatic	1.91	[0.32, 11.52]	0.481	0.721	1.08	[0.56, 2.11]	0.818	0.89
		PLWH vs PWOH in Symptomatic	8.41	[0.42, 168.18]	0.164	0.66	0.73	[0.47, 1.12]	0.151	0.452
		PLWH vs PWOH in Hospitalized	1.15	[0.14, 9.36]	0.893	0.964	1.19	[0.79, 1.8]	0.402	0.726
	SARS-CoV-2-NTD	PLWH vs PWOH in Asymptomatic	2.25	[0.42, 12.12]	0.347	0.66	1.32	[0.48, 3.64]	0.597	0.814

			Response Rate				Response Magnitudes			
Isotype	Antigen	Comparison	OR	95% CI	P-value	Q-value	GMR	95% CI	P-value	Q-value
		PLWH vs PWOH in Symptomatic	1.09	[0.25, 4.78]	0.913	0.964	0.64	[0.33, 1.24]	0.183	0.458
		PLWH vs PWOH in Hospitalized	1.04	[0.19, 5.67]	0.964	0.964	1.02	[0.56, 1.87]	0.943	0.943
	SARS-CoV-2 6P Spike	PLWH vs PWOH in Asymptomatic	2.91	[0.54, 15.54]	0.212	0.66	0.72	[0.31, 1.69]	0.448	0.726
		PLWH vs PWOH in Symptomatic	3.23	[0.47, 22.12]	0.232	0.66	0.57	[0.34, 0.95]	0.033	0.452
		PLWH vs PWOH in Hospitalized	2.37	[0.33, 17.15]	0.393	0.66	1.11	[0.68, 1.81]	0.677	0.846
IgG	CoV-2 Nucleoprotein	PLWH vs PWOH in Asymptomatic	1.56	[0.21, 11.7]	0.664	0.904	1.03	[0.31, 3.35]	0.966	0.966
		PLWH vs PWOH in Symptomatic	7.03	[0.37, 133.39]	0.194	0.78	0.52	[0.25, 1.06]	0.072	0.217
		PLWH vs PWOH in Hospitalized	1.12	[0.19, 6.57]	0.904	0.904	0.67	[0.33, 1.37]	0.278	0.468
	CoV-2 RBD	PLWH vs PWOH in Asymptomatic	1.2	[0.16, 8.96]	0.857	0.904	1.59	[0.58, 4.34]	0.364	0.468

			Response Rate				Response Magnitudes			
Isotype	Antigen	Comparison	OR	95% CI	P-value	Q-value	GMR	95% CI	P-value	Q-value
		PLWH vs PWOH in Symptomatic	6.98	[0.36, 136.77]	0.201	0.78	**0.43**	**[0.23, 0.78]**	**0.006**	**0.053**
		PLWH vs PWOH in Hospitalized	1.99	[0.25, 15.67]	0.514	0.904	0.69	[0.38, 1.26]	0.227	0.468
	CoV-2 2P Spike	PLWH vs PWOH in Asymptomatic	0.85	[0.11, 6.6]	0.873	0.904	1.73	[0.56, 5.29]	0.337	0.468
		PLWH vs PWOH in Symptomatic	5.56	[0.28, 110.24]	0.26	0.78	**0.41**	**[0.21, 0.82]**	**0.012**	**0.054**
		PLWH vs PWOH in Hospitalized	1.34	[0.17, 10.76]	0.784	0.904	0.9	[0.46, 1.75]	0.747	0.84
IgA	SARS-CoV-2 RBD	PLWH vs PWOH in Asymptomatic	3.15	[0.53, 18.58]	0.206	0.947	1.27	[0.68, 2.37]	0.454	0.68
		PLWH vs PWOH in Symptomatic	0.62	[0.17, 2.22]	0.462	0.947	0.8	[0.49, 1.31]	0.381	0.68
		PLWH vs PWOH in Hospitalized	0.95	[0.19, 4.78]	0.947	0.947	0.68	[0.46, 0.99]	0.047	0.36
	SARS-CoV-2 Nucleoprotein	PLWH vs PWOH in Asymptomatic	0.81	[0.13, 5.15]	0.825	0.947	1.2	[0.54, 2.65]	0.657	0.758

			Response Rate				Response Magnitudes			
Isotype	Antigen	Comparison	OR	95% CI	P-value	Q-value	GMR	95% CI	P-value	Q-value
		PLWH vs PWOH in Symptomatic	1.07	[0.22, 5.22]	0.93	0.947	0.76	[0.43, 1.33]	0.331	0.68
		PLWH vs PWOH in Hospitalized	0.47	[0.09, 2.37]	0.359	0.947	0.94	[0.56, 1.59]	0.821	0.821
	SARS-CoV-2 2P Spike	PLWH vs PWOH in Asymptomatic	1.74	[0.3, 9.97]	0.536	0.947	1.19	[0.68, 2.09]	0.544	0.68
		PLWH vs PWOH in Symptomatic	0.64	[0.17, 2.44]	0.511	0.947	0.94	[0.61, 1.43]	0.761	0.816
		PLWH vs PWOH in Hospitalized	0.79	[0.15, 4.13]	0.782	0.947	0.87	[0.61, 1.24]	0.443	0.68
	SARS-CoV-2-NTD	PLWH vs PWOH in Asymptomatic	3.06	[0.52, 18.06]	0.218	0.947	2.27	[0.92, 5.59]	0.074	0.37
		PLWH vs PWOH in Symptomatic	1.71	[0.36, 8.04]	0.498	0.947	0.59	[0.32, 1.11]	0.103	0.387
		PLWH vs PWOH in Hospitalized	0.81	[0.14, 4.55]	0.808	0.947	0.85	[0.51, 1.42]	0.541	0.68
	SARS-CoV-2 6P Spike	PLWH vs PWOH in Asymptomatic	1.38	[0.23, 8.23]	0.725	0.947	1.48	[0.69, 3.2]	0.318	0.68

			Response Rate				Response Magnitudes			
**Isotype**	**Antigen**	**Comparison**	**OR**	**95% CI**	**P-value**	**Q-value**	**GMR**	**95% CI**	**P-value**	**Q-value**
		PLWH vs PWOH in Symptomatic	5.92	[0.31, 113.77]	0.238	0.947	0.6	[0.37, 1]	0.048	0.36
		PLWH vs PWOH in Hospitalized	0.8	[0.15, 4.42]	0.801	0.947	0.73	[0.45, 1.19]	0.213	0.638

*: OR=Odds Ratio, +: GMR=Geometric Mean Ratio; Bold values are significant (p-value≤0.05 and q-value≤0.2). Response magnitudes include only positive responders.

Supplemental Table 3: Association of Peak COVID-19 severity with binding antibody responses adjusting for demographics, pre-existing medical conditions, smoking history, region, and days since SARS-CoV-2 diagnosis in PWOH participants

			Response Rate				Response Magnitudes			
**Isotype**	**Antigen**	**Comparison**	**OR**	**95% CI**	**P-value**	**Q-value**	**GMR**	**95% CI**	**P-value**	**Q-value**
IgG1	SARS-CoV-2 RBD	Symptomatic vs Asymptomatic in PWOH	**6.17**	**[2.27, 16.77]**	**<0.001**	**0.001**	**2.63**	**[1.7, 4.09]**	**<0.001**	**<0.001**
		Hospitalized vs Symptomatic in PWOH	2.1	[0.75, 5.88]	0.156	0.229	**2.29**	**[1.65, 3.17]**	**<0.001**	**<0.001**
	SARS-CoV-2 Nucleoprotein	Symptomatic vs Asymptomatic in PWOH	**4.72**	**[1.75, 12.69]**	**0.002**	**0.004**	**3.71**	**[2.37, 5.81]**	**<0.001**	**<0.001**
		Hospitalized vs Symptomatic in PWOH	2.11	[0.74, 5.96]	0.16	0.229	**1.59**	**[1.13, 2.22]**	**0.007**	**0.007**
	SARS-CoV-2 2P Spike	Symptomatic vs Asymptomatic in PWOH	**5.8**	**[2.04, 16.5]**	**0.001**	**0.002**	**1.84**	**[1.32, 2.55]**	**<0.001**	**<0.001**
		Hospitalized vs Symptomatic in PWOH	1.94	[0.67, 5.61]	0.218	0.273	**1.73**	**[1.35, 2.22]**	**<0.001**	**<0.001**
	SARS-CoV-2-NTD	Symptomatic vs Asymptomatic in PWOH	**60.27**	**[6.89, 526.91]**	**<0.001**	**0.001**	**5.02**	**[2.68, 9.39]**	**<0.001**	**<0.001**

			Response Rate				Response Magnitudes			
**Isotype**	**Antigen**	**Comparison**	**OR**	**95% CI**	**P-value**	**Q-value**	**GMR**	**95% CI**	**P-value**	**Q-value**
		Hospitalized vs Symptomatic in PWOH	0.88	[0.12, 6.58]	0.901	0.901	**3.03**	**[1.97, 4.66]**	**<0.001**	**<0.001**
	SARS-CoV-2 6P Spike	Symptomatic vs Asymptomatic in PWOH	**45.32**	**[5.9, 348.3]**	**<0.001**	**0.001**	**4.61**	**[2.63, 8.06]**	**<0.001**	**<0.001**
		Hospitalized vs Symptomatic in PWOH	1.17	[0.17, 8.21]	0.876	0.901	**2.23**	**[1.53, 3.26]**	**<0.001**	**<0.001**
IgG3	SARS-CoV-2 RBD	Symptomatic vs Asymptomatic in PWOH	**2.64**	**[1.2, 5.84]**	**0.016**	**0.027**	**1.57**	**[1.12, 2.2]**	**0.009**	**0.011**
		Hospitalized vs Symptomatic in PWOH	**3.43**	**[1.52, 7.71]**	**0.003**	**0.007**	**1.46**	**[1.15, 1.85]**	**0.002**	**0.003**
	SARS-CoV-2 Nucleoprotein	Symptomatic vs Asymptomatic in PWOH	**3.77**	**[1.31, 10.91]**	**0.014**	**0.027**	**1.7**	**[1.22, 2.37]**	**0.002**	**0.003**
		Hospitalized vs Symptomatic in PWOH	**2.55**	**[0.87, 7.45]**	**0.088**	**0.088**	**1.78**	**[1.38, 2.29]**	**<0.001**	**<0.001**
	SARS-CoV-2 2P Spike	Symptomatic vs Asymptomatic in PWOH	**5.31**	**[1.95, 14.47]**	**0.001**	**0.006**	**1.37**	**[1.02, 1.85]**	**0.036**	**0.04**
		Hospitalized vs Symptomatic in PWOH	**3.32**	**[1.07, 10.24]**	**0.037**	**0.051**	**1.59**	**[1.27, 1.98]**	**<0.001**	**<0.001**

Isotype	Antigen	Comparison	Response Rate				Response Magnitudes			
			OR	95% CI	P-value	Q-value	GMR	95% CI	P-value	Q-value
	SARS-CoV-2-NTD	Symptomatic vs Asymptomatic in PWOH	**3.83**	**[1.6, 9.15]**	**0.003**	**0.007**	**2.19**	**[1.4, 3.42]**	**0.001**	**0.001**
		Hospitalized vs Symptomatic in PWOH	**2.65**	**[1.04, 6.75]**	**0.041**	**0.051**	**2.51**	**[1.81, 3.47]**	**<0.001**	**<0.001**
	SARS-CoV-2 6P Spike	Symptomatic vs Asymptomatic in PWOH	**5.54**	**[2.32, 13.22]**	**<0.001**	**0.001**	1.44	[0.98, 2.12]	0.064	0.064
		Hospitalized vs Symptomatic in PWOH	**2.35**	**[0.97, 5.73]**	**0.06**	**0.066**	**1.87**	**[1.43, 2.44]**	**<0.001**	**<0.001**
IgG	CoV-2 Nucleoprotein	Symptomatic vs Asymptomatic in PWOH	3.56	[0.95, 13.35]	0.059	0.178	**2.7**	**[1.44, 5.06]**	**0.002**	**0.002**
		Hospitalized vs Symptomatic in PWOH	1.42	[0.46, 4.42]	0.542	0.542	**2.49**	**[1.64, 3.78]**	**<0.001**	**<0.001**
	CoV-2 RBD	Symptomatic vs Asymptomatic in PWOH	3.6	[0.96, 13.54]	0.058	0.178	**2.39**	**[1.4, 4.09]**	**0.001**	**0.002**
		Hospitalized vs Symptomatic in PWOH	1.48	[0.47, 4.67]	0.506	0.542	**3.53**	**[2.48, 5.03]**	**<0.001**	**<0.001**
	CoV-2 2P Spike	Symptomatic vs Asymptomatic in PWOH	3.13	[0.74, 13.29]	0.122	0.244	**3.41**	**[1.88, 6.18]**	**<0.001**	**<0.001**

			Response Rate				Response Magnitudes			
Isotype	Antigen	Comparison	OR	95% CI	P-value	Q-value	GMR	95% CI	P-value	Q-value
		Hospitalized vs Symptomatic in PWOH	1.88	[0.53, 6.7]	0.332	0.497	**3.18**	**[2.14, 4.74]**	**<0.001**	**<0.001**
IgA	SARS-CoV-2 RBD	Symptomatic vs Asymptomatic in PWOH	**5**	**[2.19, 11.41]**	**<0.001**	**0.001**	**1.53**	**[1.15, 2.06]**	**0.004**	**0.005**
		Hospitalized vs Symptomatic in PWOH	**2.33**	**[1.07, 5.07]**	**0.034**	**0.042**	**1.65**	**[1.34, 2.03]**	**<0.001**	**<0.001**
	SARS-CoV-2 Nucleoprotein	Symptomatic vs Asymptomatic in PWOH	**3.2**	**[1.29, 7.94]**	**0.012**	**0.02**	**2.71**	**[1.9, 3.85]**	**<0.001**	**<0.001**
		Hospitalized vs Symptomatic in PWOH	**3.07**	**[1.2, 7.88]**	**0.02**	**0.028**	**2.07**	**[1.58, 2.71]**	**<0.001**	**<0.001**
	SARS-CoV-2 2P Spike	Symptomatic vs Asymptomatic in PWOH	**4.03**	**[1.65, 9.85]**	**0.002**	**0.007**	**1.55**	**[1.2, 1.99]**	**0.001**	**0.001**
		Hospitalized vs Symptomatic in PWOH	**2.29**	**[0.94, 5.53]**	**0.067**	**0.074**	**1.56**	**[1.29, 1.88]**	**<0.001**	**<0.001**
	SARS-CoV-2-NTD	Symptomatic vs Asymptomatic in PWOH	**4.12**	**[1.6, 10.61]**	**0.003**	**0.007**	**1.7**	**[1.06, 2.71]**	**0.027**	**0.027**

			Response Rate				Response Magnitudes			
**Isotype**	**Antigen**	**Comparison**	**OR**	**95% CI**	**P-value**	**Q-value**	**GMR**	**95% CI**	**P-value**	**Q-value**
		Hospitalized vs Symptomatic in PWOH	**6.42**	**[2.36, 17.5]**	**<0.001**	**0.001**	**2.45**	**[1.76, 3.43]**	**<0.001**	**<0.001**
	SARS-CoV-2 6P Spike	Symptomatic vs Asymptomatic in PWOH	**4.67**	**[1.68, 12.99]**	**0.003**	**0.007**	**1.7**	**[1.22, 2.39]**	**0.002**	**0.002**
		Hospitalized vs Symptomatic in PWOH	1.64	[0.59, 4.54]	0.34	0.34	**1.85**	**[1.43, 2.39]**	**<0.001**	**<0.001**

*: OR=Odds Ratio, +: GMR=Geometric Mean Ratio; Bold values are significant (p-value≤0.05 and q-value≤0.2). Response magnitudes include only positive responders

Supplemental Table 4: Association of Peak COVID-19 severity with binding antibody responses adjusting for demographics, pre-existing medical conditions, smoking history, region, and days since SARS-CoV-2 diagnosis in PLWH

			Response Rate				Response Magnitudes			
**Isotype**	**Antigen**	**Comparison**	**OR**	**95% CI**	**P-value**	**Q-value**	**GMR**	**95% CI**	**P-value**	**Q-value**
IgG1	SARS-CoV-2 RBD	Symptomatic vs Asymptomatic in PLWH	**34.89**	**[1.77, 688.11]**	**0.02**	**0.065**	1.08	[0.54, 2.15]	0.836	0.836
		Hospitalized vs Symptomatic in PLWH	3.07	[0.03, 271.63]	0.624	0.775	**12.77**	**[3.62, 45.03]**	**<0.001**	**<0.001**
	SARS-CoV-2 Nucleoprotein	Symptomatic vs Asymptomatic in PLWH	**39.14**	**[1.98, 774.7]**	**0.016**	**0.065**	1.43	[0.7, 2.91]	0.33	0.472
		Hospitalized vs Symptomatic in PLWH	0.47	[0.01, 21.36]	0.697	0.775	**6.25**	**[1.69, 23.09]**	**0.006**	**0.012**
	SARS-CoV-2 2P Spike	Symptomatic vs Asymptomatic in PLWH	**28.52**	**[1.49, 547.21]**	**0.026**	**0.066**	1.47	[0.87, 2.5]	0.153	0.255

			Response Rate				Response Magnitudes			
Isotype	Antigen	Comparison	OR	95% CI	P-value	Q-value	GMR	95% CI	P-value	Q-value
		Hospitalized vs Symptomatic in PLWH	0.76	[0.02, 37.51]	0.889	0.889	**3.89**	**[1.48, 10.23]**	**0.006**	**0.012**
	SARS-CoV-2-NTD	Symptomatic vs Asymptomatic in PLWH	**7.95**	**[1.05, 60.29]**	**0.045**	**0.09**	1.14	[0.46, 2.84]	0.784	0.836
		Hospitalized vs Symptomatic in PLWH	10.36	[0.31, 348.67]	0.193	0.321	**31.39**	**[6.75, 145.92]**	**<0.001**	**<0.001**
	SARS-CoV-2 6P Spike	Symptomatic vs Asymptomatic in PLWH	**54.03**	**[2.44, 1194.36]**	**0.012**	**0.065**	1.17	[0.53, 2.55]	0.7	0.836
		Hospitalized vs Symptomatic in PLWH	7.97	[0.11, 567.01]	0.34	0.486	**16.09**	**[4.22, 61.36]**	**<0.001**	**<0.001**
IgG3	SARS-CoV-2 RBD	Symptomatic vs Asymptomatic in PLWH	**7.44**	**[1.52, 36.46]**	**0.013**	**0.045**	1.08	[0.64, 1.82]	0.768	0.854
		Hospitalized vs Symptomatic in PLWH	6.82	[0.38, 122.66]	0.193	0.296	2.45	[0.94, 6.34]	0.066	0.164

			Response Rate				Response Magnitudes			
Isotype	Antigen	Comparison	OR	95% CI	P-value	Q-value	GMR	95% CI	P-value	Q-value
	SARS-CoV-2 Nucleoprotein	Symptomatic vs Asymptomatic in PLWH	**25.18**	**[1.2, 526.8]**	**0.038**	**0.094**	1.15	[0.68, 1.96]	0.605	0.756
		Hospitalized vs Symptomatic in PLWH	0.68	[0.01, 37.44]	0.851	0.945	**4.21**	**[1.58, 11.23]**	**0.004**	**0.04**
	SARS-CoV-2 2P Spike	Symptomatic vs Asymptomatic in PLWH	**44.62**	**[2.22, 898.4]**	**0.013**	**0.045**	1	[0.62, 1.6]	0.998	0.998
		Hospitalized vs Symptomatic in PLWH	0.87	[0.02, 44.98]	0.945	0.945	**2.81**	**[1.18, 6.66]**	**0.019**	**0.064**
	SARS-CoV-2-NTD	Symptomatic vs Asymptomatic in PLWH	4.16	[0.94, 18.52]	0.061	0.122	1.39	[0.67, 2.9]	0.373	0.622
		Hospitalized vs Symptomatic in PLWH	5.7	[0.38, 85.06]	0.207	0.296	**5.3**	**[1.41, 19.86]**	**0.013**	**0.064**

			Response Rate				Response Magnitudes			
Isotype	Antigen	Comparison	OR	95% CI	P-value	Q-value	GMR	95% CI	P-value	Q-value
	SARS-CoV-2 6P Spike	Symptomatic vs Asymptomatic in PLWH	**17.91**	**[2.58, 124.26]**	**0.004**	**0.035**	0.82	[0.45, 1.47]	0.495	0.708
		Hospitalized vs Symptomatic in PLWH	5.01	[0.22, 114.8]	0.313	0.391	2.62	[0.88, 7.79]	0.082	0.164
IgG	CoV-2 Nucleoprotein	Symptomatic vs Asymptomatic in PLWH	**25.06**	**[1.22, 515.38]**	**0.037**	**0.115**	1.4	[0.59, 3.32]	0.441	0.529
		Hospitalized vs Symptomatic in PLWH	0.35	[0.01, 16.6]	0.596	0.738	3.32	[0.75, 14.69]	0.114	0.227
	CoV-2 RBD	Symptomatic vs Asymptomatic in PLWH	**25.16**	**[1.19, 533.32]**	**0.038**	**0.115**	1.02	[0.49, 2.12]	0.959	0.959
		Hospitalized vs Symptomatic in PLWH	0.51	[0.01, 27.31]	0.738	0.738	**9.15**	**[2.58, 32.45]**	**0.001**	**0.002**

			Response Rate				Response Magnitudes			
**Isotype**	**Antigen**	**Comparison**	**OR**	**95% CI**	**P-value**	**Q-value**	**GMR**	**95% CI**	**P-value**	**Q-value**
	CoV-2 2P Spike	Symptomatic vs Asymptomatic in PLWH	17.4	[0.8, 379.67]	0.069	0.139	1.41	[0.62, 3.19]	0.407	0.529
		Hospitalized vs Symptomatic in PLWH	0.38	[0.01, 20.77]	0.637	0.738	**11.9**	**[2.86, 49.48]**	**0.001**	**0.002**
IgA	SARS-CoV-2 RBD	Symptomatic vs Asymptomatic in PLWH	3.1	[0.83, 11.53]	0.091	0.221	1.23	[0.73, 2.08]	0.439	0.548
		Hospitalized vs Symptomatic in PLWH	11.17	[0.78, 159.99]	0.075	0.221	1.77	[0.77, 4.1]	0.181	0.259
	SARS-CoV-2 Nucleoprotein	Symptomatic vs Asymptomatic in PLWH	3.44	[0.69, 17.19]	0.133	0.221	**2.05**	**[1.14, 3.7]**	**0.017**	**0.061**
		Hospitalized vs Symptomatic in PLWH	1.08	[0.07, 18.01]	0.955	0.955	**3.08**	**[1.06, 8.94]**	**0.038**	**0.096**

			Response Rate				Response Magnitudes			
Isotype	Antigen	Comparison	OR	95% CI	P-value	Q-value	GMR	95% CI	P-value	Q-value
	SARS-CoV-2 2P Spike	Symptomatic vs Asymptomatic in PLWH	2.57	[0.66, 10]	0.173	0.247	1.45	[0.93, 2.27]	0.103	0.207
		Hospitalized vs Symptomatic in PLWH	4.93	[0.34, 71.7]	0.243	0.304	1.72	[0.81, 3.65]	0.156	0.259
	SARS-CoV-2-NTD	Symptomatic vs Asymptomatic in PLWH	**7.05**	**[1.45, 34.29]**	**0.016**	**0.137**	1.01	[0.51, 1.98]	0.984	0.984
		Hospitalized vs Symptomatic in PLWH	9.27	[0.57, 151.61]	0.118	0.221	**8.01**	**[2.55, 25.13]**	**<0.001**	**0.004**
	SARS-CoV-2 6P Spike	Symptomatic vs Asymptomatic in PLWH	**27.62**	**[1.45, 526.96]**	**0.027**	**0.137**	1.03	[0.6, 1.77]	0.917	0.984
		Hospitalized vs Symptomatic in PLWH	0.31	[0.01, 13.22]	0.538	0.598	**3.33**	**[1.22, 9.04]**	**0.018**	**0.061**

[*]: OR=Odds Ratio, [+]: GMR=Geometric Mean Ratio; Bold values are significant (p-value≤0.05 and q-value≤0.2). Response magnitudes include only positive responders

Supplemental Table 5: Association of HIV serostatus and COVID19 severity with ACE-2 blocking to SARS-CoV-2 spike responses adjusting for COVID-19 severity (in the first row only), demographics, pre-existing medical conditions, smoking history, region, and days since SARS-CoV-2 diagnosis

		Response Rate			Percent Blocking Among Positive Responders		
**Participants**	**Comparison**	**OR***	**95% CI**	**P-value**	**OR***	**95% CI**	**P-value**
All	PLWH vs PWOH	0.67	[0.08, 6.12]	0.708	0.56	[0.22, 1.4]	0.219
All	PLWH vs PWOH in Asymptomatic	0.9	[0.07, 12.07]	0.935	2.11	[0.19, 22.98]	0.54
	PLWH vs PWOH in Symptomatic	0.55	[0.07, 4.14]	0.564	0.32	[0.07, 1.53]	0.153
	PLWH vs PWOH in Hospitalized	0.72	[0.03, 20.08]	0.844	0.73	[0.22, 2.49]	0.617
PWOH	Symptomatic vs Asymptomatic	2.78	[0.26, 29.35]	0.396	4.94	[0.8, 30.63]	0.086
	Hospitalized vs Symptomatic	6.56	[0.48, 88.89]	0.157	**3.37**	**[1.43, 7.94]**	**0.005**
PLWH	Symptomatic vs Asymptomatic	1.54	[0.13, 18.35]	0.735	1.59	[0.18, 13.83]	0.674
	Hospitalized vs Symptomatic	7.62	[0.16, 371.69]	0.306	16.18	[0.82, 320.83]	0.068

*: OR=Odds Ratio, Bold values are significant (p-value≤0.05).

Supplemental Table 6: Spearman Correlation of CD4 Counts with Immune Responses[a]

Isotype-Antigen	Correlation Coefficient	P-value
IgA.Nucleoprotein	0.13	0.52
IgA.NTD	-0.05	0.83
IgA.SARS-CoV-2 RBD	0.02	0.91
IgA.2P Spike	-0.02	0.92
IgA.6P Spike	-0.11	0.58
IgG1.Nucleoprotein	0.07	0.75
IgG1.NTD	-0.19	0.35
IgG1.SARS-CoV-2 RBD	-0.22	0.27
IgG1.2P Spike	-0.19	0.34
IgG1.6P Spike	-0.25	0.22
IgG3.Nucleoprotein	-0.02	0.93
IgG3.NTD	-0.16	0.42
IgG3.SARS-CoV-2 RBD	-0.21	0.29
IgG3.2P Spike	-0.13	0.53
IgG3.6P Spike	-0.25	0.21
MSD IgG.CoV-2 Nucleocapsid	0.09	0.66
MSD IgG.SARS-CoV-2 RBD	-0.22	0.28
MSD IgG.2P Spike	-0.23	0.26

ACE2.2P Spike	-0.25	0.22

[a]26 PLWH had recently available CD4 count data. Sigificant correlations were defined as $p \leq 0.05$.

Supplemental Table 7: SARS-CoV-2-specific Immune Responses Stratified by HIV Viral Load Detection Status[a]

Isotype	Antigen	With Detectable VL<br>Response Rate(95% CI)	With undetectable VL<br>Response Rate(95% CI)	Fisher's Exact Test P-value
ACE2	2P Spike	2/3 = 66.7% (20.8%, 93.8%)	21/24 = 87.5% (69.0%, 95.7%)	0.395
IgA	Nucleoprotein	2/3 = 66.7% (20.8%, 93.8%)	21/24 = 87.5% (69.0%, 95.7%)	0.395
	NTD	3/3 = 100.0% (43.9%, 100.0%)	20/24 = 83.3% (64.1%, 93.3%)	1
	SARS-CoV-2 RBD	3/3 = 100.0% (43.9%, 100.0%)	19/24 = 79.2% (59.5%, 90.8%)	1
	2P Spike	3/3 = 100.0% (43.9%, 100.0%)	20/24 = 83.3% (64.1%, 93.3%)	1
	6P Spike	3/3 = 100.0% (43.9%, 100.0%)	22/24 = 91.7% (74.2%, 97.7%)	1
IgG1	Nucleoprotein	3/3 = 100.0% (43.9%, 100.0%)	22/24 = 91.7% (74.2%, 97.7%)	1
	NTD	3/3 = 100.0% (43.9%, 100.0%)	21/24 = 87.5% (69.0%, 95.7%)	1
	SARS-CoV-2 RBD	3/3 = 100.0% (43.9%, 100.0%)	23/24 = 95.8% (79.8%, 99.3%)	1
	2P Spike	3/3 = 100.0% (43.9%, 100.0%)	22/24 = 91.7% (74.2%, 97.7%)	1
	6P Spike	3/3 = 100.0% (43.9%, 100.0%)	22/24 = 91.7% (74.2%, 97.7%)	1
IgG3	Nucleoprotein	3/3 = 100.0% (43.9%, 100.0%)	23/24 = 95.8% (79.8%, 99.3%)	1
	NTD	3/3 = 100.0% (43.9%, 100.0%)	19/24 = 79.2% (59.5%, 90.8%)	1

	SARS-CoV-2 RBD	3/3 = 100.0% (43.9%, 100.0%)	21/24 = 87.5% (69.0%, 95.7%)	1
	2P Spike	3/3 = 100.0% (43.9%, 100.0%)	23/24 = 95.8% (79.8%, 99.3%)	1
	6P Spike	3/3 = 100.0% (43.9%, 100.0%)	21/24 = 87.5% (69.0%, 95.7%)	1
MSD IgG	SARS-CoV-2 RBD	3/3 = 100.0% (43.9%, 100.0%)	23/24 = 95.8% (79.8%, 99.3%)	1
	2P Spike	3/3 = 100.0% (43.9%, 100.0%)	23/24 = 95.8% (79.8%, 99.3%)	1
	CoV-2 Nucleocapsid	3/3 = 100.0% (43.9%, 100.0%)	22/24 = 91.7% (74.2%, 97.7%)	1

[a]27 PLWH had recently available VL data. Detectable VL was defined as ≥50 copies/mL and undetectable VL<50 copies/mL. Sigificant correlations were defined as $p \leq 0.05$.

Supplemental Table 8: Association of HIV serostatus and COVID19 severity with ADCP response to SARS-CoV-2 6P Spike adjusting for demographics, pre-existing medical conditions, smoking history, region, and days since SARS-CoV-2 diagnosis

Participants	Comparison	Response Rate			Response Magnitude among Positive Responders		
		OR*	95% CI	P-value	GMR+	95% CI	P-value
All	PLWH vs PWOH	0.76	[0.28, 2.3]	0.614	0.86	[0.72, 1.03]	0.106
All	PLWH vs PWOH in Asymptomatic	0.81	[0.13, 4.84]	0.814	0.77	[0.5, 1.19]	0.234
	PLWH vs PWOH in Symptomatic	4.35	[0.23, 83.54]	0.33	**0.77**	**[0.59, 0.99]**	**0.045**
	PLWH vs PWOH in Hospitalized	**0.23**	**[0.06, 0.93]**	**0.039**	1.02	[0.78, 1.33]	0.894
PWOH	Symptomatic vs Asymptomatic	**4.44**	**[1.72, 11.44]**	**0.002**	1.11	[0.93, 1.33]	0.236
	Hospitalized vs Symptomatic	2.19	[0.77, 6.19]	0.142	**1.21**	**[1.07, 1.38]**	**0.003**
PLWH	Symptomatic vs Asymptomatic	**19.3**	**[1.01, 367.69]**	**0.049**	0.85	[0.64, 1.14]	0.275
	Hospitalized vs Symptomatic	0.09	[0, 3.37]	0.195	1.24	[0.71, 2.17]	0.449

*: OR=Odds Ratio, +: GMR=Geometric Mean Ratio; Bold values are significant (p-value≤0.05).

Supplemental Table 9: Sensitivity analysis: Comparison of models adjusting for either the full list of factors (Primary model: demographics, pre-existing medical conditions, smoking history, region, and days since SARS-CoV-2 diagnosis) or a truncated list (Sensitivity analysis model: COVID-19 severity (except where this factor is the comparison), days since SARS-CoV-2 diagnosis, age, sex assigned at birth, and region). Statistical differences are highlighted in the tables below with addition information in the subheadings. *: OR=Odds Ratio, +: GMR=Geometric Mean Ratio; Bold values are significant (p-value≤0.05 and q-value≤0.2)

			**Sensitivity Analysis**				**Primary Analysis**			
Binding Ab response rates comparison by HIV infection status (PLWH vs PWOH uninfected) overall										
**Isotype**	**Antigen**	**Participants**	**OR***	**95% CI**	**P-value**	**Q-value**	**OR***	**95% CI**	**P-value**	**Q-value**
**IgG1**	SARS-CoV-2 RBD	All	**4.28**	**[1.19, 23.32]**	**0.023**	**0.117**	3.65	[0.97, 20.51]	0.057	0.245
**IgG3**	SARS-CoV-2 RBD	All	2.09	[0.85, 5.91]	0.114	0.19	**2.81**	**[1.05, 8.68]**	**0.039**	**0.097**
	SARS-CoV-2 6P Spike	All	2.29	[0.84, 7.34]	0.11	0.19	**3.23**	**[1.09, 11.39]**	**0.033**	**0.097**
Binding Ab response magnitudes comparison by HIV infection status (PLWH vs PWOH uninfected) overall										
**Isotype**	**Antigen**	**Participants**	**GMR+**	**95% CI**	**P-value**	**Q-value**	**GMR+**	**95% CI**	**P-value**	**Q-value**
**IgG1**	SARS-CoV-2 RBD	All	**0.66**	**[0.44, 0.99]**	**0.046**	**0.116**	0.75	[0.5, 1.14]	0.184	0.307
	SARS-CoV-2 6P Spike	All	**0.64**	**[0.41, 0.98]**	**0.042**	**0.116**	**0.63**	**[0.4, 1]**	**0.05**	**0.138**
**IgG**	CoV-2 RBD	All	0.67	[0.45, 1.01]	0.058	0.173	**0.63**	**[0.42, 0.96]**	**0.031**	**0.093**
Binding Ab response magnitudes comparison by HIV infection status (PLWH vs PWOH uninfected) stratified by peak COVID-19 severity										
**Isotype**	**Antigen**	**Participants**	**GMR+**	**95% CI**	**P-value**	**Q-value**	**GMR+**	**95% CI**	**P-value**	**Q-value**
**IgG1**	SARS-CoV-2 RBD	Asymptomatic	1.83	[0.69, 4.84]	0.227	0.496	**2.7**	**[1.02, 7.15]**	**0.046**	**0.138**
		Symptomatic	**0.35**	**[0.19, 0.67]**	**0.001**	**0.008**	**0.41**	**[0.22, 0.77]**	**0.005**	**0.02**
	SARS-CoV-2 Nucleoprotein	Symptomatic	**0.39**	**[0.21, 0.74]**	**0.004**	**0.014**	**0.38**	**[0.2, 0.74]**	**0.004**	**0.02**
	SARS-CoV-2-NTD	Symptomatic	**0.27**	**[0.12, 0.61]**	**0.002**	**0.008**	**0.23**	**[0.1, 0.51]**	**<0.001**	**0.003**
	SARS-CoV-2 6P Spike	Symptomatic	**0.26**	**[0.13, 0.49]**	**<0.001**	**0.001**	**0.25**	**[0.13, 0.5]**	**<0.001**	**0.001**
**IgG**	CoV-2 RBD	Symptomatic	**0.41**	**[0.22, 0.76]**	**0.004**	**0.028**	**0.43**	**[0.23, 0.78]**	**0.006**	**0.053**
	CoV-2 2P Spike	Symptomatic	**0.38**	**[0.19, 0.76]**	**0.006**	**0.028**	**0.41**	**[0.21, 0.82]**	**0.012**	**0.054**
Binding Ab response rates comparison between peak COVID-19 severity stratified by HIV infection status										

Isotype	Antigen	Comparison	OR*	95% CI	P-value	Q-value	OR*	95% CI	P-value	Q-value
**IgG**	CoV-2 Nucleoprotein	Symptomatic vs Asymptomatic in PWOH	**4.85**	**[1.43, 16.48]**	**0.011**	**0.04**	3.56	[0.95, 13.35]	0.059	0.178
	CoV-2 RBD	Symptomatic vs Asymptomatic in PWOH	**4.72**	**[1.38, 16.1]**	**0.013**	**0.04**	3.6	[0.96, 13.54]	0.058	0.178
	CoV-2 2P Spike	Symptomatic vs Asymptomatic in PWOH	**4.09**	**[1.07, 15.64]**	**0.04**	**0.079**	3.13	[0.74, 13.29]	0.122	0.244
**IgG**	CoV-2 Nucleoprotein	Symptomatic vs Asymptomatic in PLWH	16.1	[0.79, 328.28]	0.071	0.218	**25.06**	**[1.22, 515.38]**	**0.037**	**0.115**
	CoV-2 RBD	Symptomatic vs Asymptomatic in PLWH	15.82	[0.78, 322.63]	0.073	0.218	**25.16**	**[1.19, 533.32]**	**0.038**	**0.115**

Binding Ab response magnitudes comparison between peak COVID-19 severity stratified by HIV infection status

Isotype	Antigen	Comparison	GMR+	95% CI	P-value	Q-value	GMR+	95% CI	P-value	Q-value
**IgG**	CoV-2 Nucleoprotein	Hospitalized vs Symptomatic in PLWH	**5.15**	**[1.2, 22.18]**	**0.028**	**0.056**	3.32	[0.75, 14.69]	0.114	0.227
	CoV-2 RBD	Hospitalized vs Symptomatic in PLWH	**12.23**	**[3.46, 43.25]**	**<0.001**	**<0.001**	**9.15**	**[2.58, 32.45]**	**0.001**	**0.002**
	CoV-2 2P Spike	Hospitalized vs Symptomatic in PLWH	**16.88**	**[4.07, 69.95]**	**<0.001**	**<0.001**	**11.9**	**[2.86, 49.48]**	**0.001**	**0.002**

Percent ACE-2 receptor blocking comparison between peak COVID-19 severity stratified by HIV infection status

Marker	Participants	Comparison	OR*	95% CI	P-value	OR*	95% CI	P-value
**ACE-2**	PWOH	Hospitalized vs Symptomatic	**3.18**	**[1.35, 7.52]**	**0.008**	**3.37**	**[1.43, 7.94]**	**0.005**
	PLWH	Hospitalized vs Symptomatic	**18.32**	**[0.99, 340.46]**	**0.051**	16.18	[0.82, 320.83]	0.068

ADCP response rates comparison by HIV infection status and peak COVID-19 severity

Marker	Participants	Comparison	OR*	95% CI	P-value	OR*	95% CI	P-value
**ADCP**	Hospitalized	PLWH vs PWOH	**0.23**	**[0.06, 0.91]**	**0.036**	**0.23**	**[0.06, 0.93]**	**0.039**
	PWOH	Symptomatic vs Asymptomatic	**4.53**	**[1.82, 11.3]**	**0.001**	**4.44**	**[1.72, 11.44]**	**0.002**
	PLWH	Symptomatic vs Asymptomatic	17.29	[0.9, 331.01]	0.058	**19.3**	**[1.01, 367.69]**	**0.049**

ADCP response magnitudes comparison by HIV infection status and peak COVID-19 severity								
**Marker**	Participants	**Comparison**	**GMR+**	**95% CI**	**P-value**	**GMR+**	**95% CI**	**P-value**
**ADCP**	Symptomatic	PLWH vs PWOH	0.78	[0.6, 1.01]	0.061	**0.77**	**[0.59, 0.99]**	**0.045**
	PWOH	Hospitalized vs Symptomatic	**1.23**	**[1.08, 1.4]**	**0.001**	**1.21**	**[1.07, 1.38]**	**0.003**
	PLWH	Hospitalized vs Symptomatic	1.24	[0.71, 2.17]	0.447	1.24	[0.71, 2.17]	0.449

Supplemental Table 10: BAMA and ADCP antigen panels

Antigen Name (short)	Antigen Name (full)	Source
RBD	SARS-CoV-2 DB-His	Neil King/Lauren Baker, Institute for Protein Design, Seattle, Washington
2P Spike	SARS-CoV-2 spike (PreS Nexelis)	Nexelis
2P Spike	2019-nCoV S/293F	Duke Protein Production Facility
6P Spike	SARS-CoV-2 S Hexapro_293F_PPF	Duke Protein Production Facility
Nucleoprotein	SARS-CoV-2 Nucleoprotein His	Cedarlane, Burlington, NC; Cat# REC31812-500
NTD	SARS-CoV-2-NTD-AVI Biotin	NIH/NIAID/VRC, Peter Kwong
